# Evaluating a mobile-based intervention to promote the mental health of informal dementia caregivers in Singapore: Study protocol for a pilot two-armed randomised controlled trial

**DOI:** 10.1371/journal.pone.0305729

**Published:** 2024-06-24

**Authors:** Yun Ting Lee, Qi Yuan, YunJue Zhang, Ellaisha Samari, Richard Goveas, Li Ling Ng, Mythily Subramaniam

**Affiliations:** 1 Research Division, Institute of Mental Health, Singapore, Singapore; 2 Department of Geriatric Psychiatry, Institute of Mental Health, Singapore, Singapore; 3 Department of Psychological Medicine, Changi General Hospital, Singapore, Singapore; University Hospital Cologne: Uniklinik Koln, GERMANY

## Abstract

**Background:**

Informal caregivers of persons with dementia (PWD) often suffer adverse impacts on their mental health and require interventions for effective support. As they are often occupied with providing care, web-based interventions could be more convenient and efficient for them. However, there is currently a dearth of evidence-based mobile interventions to enhance the mental well-being of dementia caregivers locally, especially ones that are user-centered and culturally relevant. Hence, having designed an app based on feedback from local dementia caregivers, this study will evaluate the effectiveness of this mobile app in promoting the mental health of informal caregivers of PWD in Singapore.

**Methods:**

A pilot two-armed randomised controlled trial will be conducted on 60 informal caregivers of PWD recruited via convenience and snowball sampling. Thirty participants will be assigned to the intervention group, while another 30 will be in a waiting-list control group. Questionnaires will be administered at baseline and one month after, with the primary outcome being the difference in the change of depressive symptoms among the two groups.

**Statistical analysis:**

Primary analyses will follow the intention-to-treat principle and compare changes from baseline to the one-month follow-up time point relative to the control group. A repeated measures ANOVA will be conducted to examine differences between the groups over time.

**Significance:**

To our knowledge, this is the first study in Singapore that seeks to promote the mental health of informal dementia caregivers through a mobile-based intervention. The findings can inform the development and evaluation of future evidence-based digital interventions for local informal caregivers of PWD to address the gap in availability of such resources for them.

**Trial registration:**

ClinicalTrials.gov (NCT05551533). Registration date: September 22, 2022.

## Background

Based on a nationwide study conducted in 2013, approximately 10% of older adults above the age of 60 years suffer from dementia in Singapore [1], and this number will likely grow due to the rapidly ageing population [2]. With this, the cost of informal dementia care is also expected to rise [3] as more people will have to take on the role of caring for PWD living at home. As dementia progresses, PWD require high levels of care which is mostly provided by informal caregivers, usually family members of the PWD [4] who are often untrained for this highly demanding role [5].

As a result, informal dementia caregivers often face physical, financial, and social stressors which increase their risk of poor mental health outcomes [6]. The transactional model of stress and coping [7] is commonly used to understand this stress experienced by dementia caregivers [8]. The model posits that stress is a response experienced after a two-stage appraisal process by the individual. While the primary appraisal involves the interpretation of the stressor based on one’s background, past experiences, and personality, the secondary appraisal is the assessment of available coping resources and strategies to manage the stressor [9]. Accordingly, caregivers appraise a situation as stressful when the demands of the stressor exceed the available coping resources [10]. Consequently, this strain may manifest as emotional and psychological health outcomes such as psychological morbidity [4, 11].

High rates of caregiver burden and psychological morbidity (i.e., depression, anxiety) amongst informal dementia caregivers are well-documented in the extant literature. For instance, Connors et al. [12] found that 47.4% of caregivers of PWD had clinically significant levels of burden at baseline, with this proportion increasing to 56.8% after three years. Similarly, a recent meta-analysis reported a pooled prevalence of 49.3% and 31.2% amongst caregivers of PWD for burden and depression, respectively [13]. Likewise, a meta-analysis of 17 studies found that the aggregate prevalence of depression and anxiety amongst caregivers of patients with Alzheimer’s disease was 34.0% and 43.6% respectively [14]. In the Asian context, a 42.8% prevalence of depression was reported for a sample of caregivers of PWD in China [15], while 64.6% of a small sample of dementia caregivers in Hong Kong were clinically at risk for depression [16]. Taken together, the existing literature indicates significant rates of mental health concerns amongst informal dementia caregivers, which requires intervention.

### Protective factors for dementia caregiving

Certain protective factors have been shown to buffer negative outcomes for informal caregivers. Notably, prior research revealed that higher caregiving self-efficacy, which refers to the caregiver’s perception of their ability to perform caregiving tasks competently [17], is associated with lower levels of depressive symptoms [18] and mediates caregiver burden to improve psychological well-being [19]. Similarly, caregivers’ knowledge of dementia can help them build confidence in managing PWD, reducing caregiver burden and anxiety [20].

Another factor which could determine the extent of stress caregivers experience is coping strategies [21], with Lazarus and Folkman’s model [7] postulating that the type of coping strategy employed mediates the individual’s adaptation to stress. To elaborate, emotion-focused coping strategies have been shown to reduce caregiver anxiety, depression, burnout, and stress, while problem-focused coping strategies are linked to larger improvements in depressive symptoms, anxiety, and perceived stress [22]. Additionally, identifying the positive aspects of caregiving (PAC) is another protective factor for dementia caregiving, with a higher perceived PAC associated with fewer depressive symptoms and lower burden [23]. Overall, interventions to enhance these areas may help to protect the mental health of informal dementia caregivers.

### Mobile-based interventions

For informal caregivers who are largely occupied with caregiving duties, have transportation difficulties, or do not want to leave the PWD unattended, in-person interventions may be too time-consuming and burdensome [24]. For instance, informal caregivers of PWD in Singapore reported an average of 55 hours spent weekly on caregiving [25]. For this reason, the use of mobile devices in healthcare is beneficial as they are highly accessible, personal, and cost-effective [26, 27]. In addition, access to smartphones has become increasingly widespread, with 97% of Singapore residents owning a smartphone [28]. Furthermore, local caregivers of PWD in our prior study expressed interest and receptivity towards using digital health interventions provided they include helpful features to assist them in caring for PWD or enhancing their well-being [29]. Some of these functions mentioned by caregivers of PWD in the study include knowledge on dementia, caring for PWD, and self-care, and a list of resources [29]. In view of these, there is immense potential for mobile interventions to be a more viable way of supporting informal caregivers in the local setting. In fact, there has already been a proliferation of mobile health interventions in Western countries due to their convenience, privacy-preserving delivery, and ubiquitous nature [30]. Empirical evidence also suggests that the use of mobile apps appears to be a feasible assistive technology intervention for caregivers of PWD [31] and potentially effective in improving a range of psychological outcomes such as reducing levels of burden, anxiety, and depression [32–34].

However, few of the apps presently available in the market have been developed specifically to address the needs of informal caregivers of PWD. A recent review by Castillo et al. [35] found only 16 apps which met their criteria for a focus on dementia care for informal caregivers of PWD, out of which only 3 were supported by some evidence in the literature. In another review, Brown et al. [36] identified only 5 out of 13 apps which had a focus on caregivers and all these apps had limited functions to meet the complex needs of caregivers of PWD. Lastly, a review by Rathnayake et al. [37] revealed a lack of rigorously designed studies with a clear theoretical framework and user-centered approach to app design. Findings from the above studies concurred that the apps reviewed were insufficient to address the multidimensional needs of caregivers of PWD and highlighted the need to develop more user-centered and evidence-based apps [35].

More importantly, none of the apps reviewed are based in Singapore. To the best of our knowledge, there is currently only one mobile app developed to support dementia caregivers in Singapore (i.e., CARA). Key features of the CARA app include (1) helping to locate and enable the safe return of missing PWD, (2) involving and notifying other family members by connecting with them through a connected care circle, (3) providing tailored discounts and privileges for members, and (4) access to a list of solution providers for various needs [38]. This highlights a clear gap in the development and empirical evaluation of mobile-based interventions with culturally sensitive resources for different stages of dementia in Singapore. This study will therefore evaluate the effectiveness of a multi-component mobile-based intervention comprising culturally relevant knowledge and resources aimed at improving mental health outcomes for informal caregivers of PWD in Singapore. We aim to examine differences in levels of depressive and anxiety symptoms, caregiver burden, mental well-being, caregiving self-efficacy, knowledge of dementia, positive aspects of caregiving, and coping strategies before and post-intervention, as well as between the intervention and control groups.

## Methods

### Study design

A pilot two-armed randomized control trial (RCT) design will be adopted to test the effectiveness of the *Kampung Care* app (*Kampung Care* is a local expression of a caring community). Sixty eligible caregivers of PWD will be enrolled in the RCT and randomly allocated to either the intervention or control group. No blinding will be used as participants and study team members need to be aware of which group the participant belongs to in order to start the intervention. Participants in the intervention group will use the app for one month, while those in the control group will be on a waiting list for one month. Outcome measures will be assessed at baseline and one-month after the baseline assessment (post-assessment). Thereafter, qualitative interviews will be conducted with participants from the intervention group to gather feedback on their experience with the app and areas for improvement. The Standard Protocol Items: Recommendations for Interventional Trials (SPIRIT) checklist is provided (see S1 File).

### Inclusion and exclusion criteria

Participants will be included in the study if they: (1) are at least 21 years of age; (2) a Singapore citizen or permanent resident; (3) currently taking care of a PWD; (4) able to read, write, and speak in English (i.e., the language used for the intervention); and (3) proficient in using mobile applications. Due to ethical considerations, caregivers who are pregnant or have vision or hearing problems will be excluded.

### Sample size

Given that this is a pilot RCT with the primary aim of assessing the feasibility and potential effectiveness of the ’Kampung Care’ mobile intervention, we followed the recommendation from a methodological study that a sample size of 25 per intervention arm is required if the main study is designed with 90% power and two-sided 5% significance for an intervention with a small effect size [39]. No formula or software was used in the calculation. With a 1:1 allocation ratio, the required sample size was determined to be 50. As mobile-based intervention studies tend to have high attrition rates, we doubled our preliminary sample size to 100 to ensure there will be a sufficient sample for analysis. However, following the initial recruitment, we found the attrition rate among our target group to be quite low and thus adjusted our final sample to 60 –with 30 participants randomly allocated to the intervention group and waitlist control group respectively. For the in-depth interview, we intend to recruit up to 20 participants from the intervention group. This is from an experiential perspective. However, the final sample will depend on data saturation–the point at which no new information is being observed and collected to form distinct themes.

### Participants

Participants will be referred by collaborating clinicians from the outpatient clinic of the Institute of Mental Health (a tertiary mental health service provider) and a geriatric clinic of a local hospital when they accompany the PWD during outpatient visits. Recruitment flyers may be put up in clinics as posters and/or distributed to clinicians to pass to potential participants interested in the study to contact us. In addition, we will also contact caregivers who have participated in our prior studies. Snowball recruitment will also be used by asking all caregivers with whom we come into contact to refer other potential participants. Collaborating clinicians and caregivers who refer eligible participants to us will be reminded to obtain approval from the potential participant first. Thereafter, the study team will either follow up with the potential participant to obtain their contact details at the clinic or directly contact them via phone call. Public advertisement of the study will be done through social media platforms like Facebook and LinkedIn. Lastly, we will also approach external organisations with a large base of caregivers of PWD to ask for their help in advertising the study online.

### Study procedure

Once a caregiver agrees to participate, a study team member will schedule a session with them. All sessions will be conducted either online via a video-conferencing platform like Zoom or in-person, based on the caregiver’s preference. After the study team member goes through the informed consent form and obtains the participant’s signed consent, screening will be done using the 4-item version of the Zarit Burden Interview (ZBI) [40]. Caregivers who score 4 and above will be included in the study, while those who score 3 and below will be excluded.

**Fig 1:**
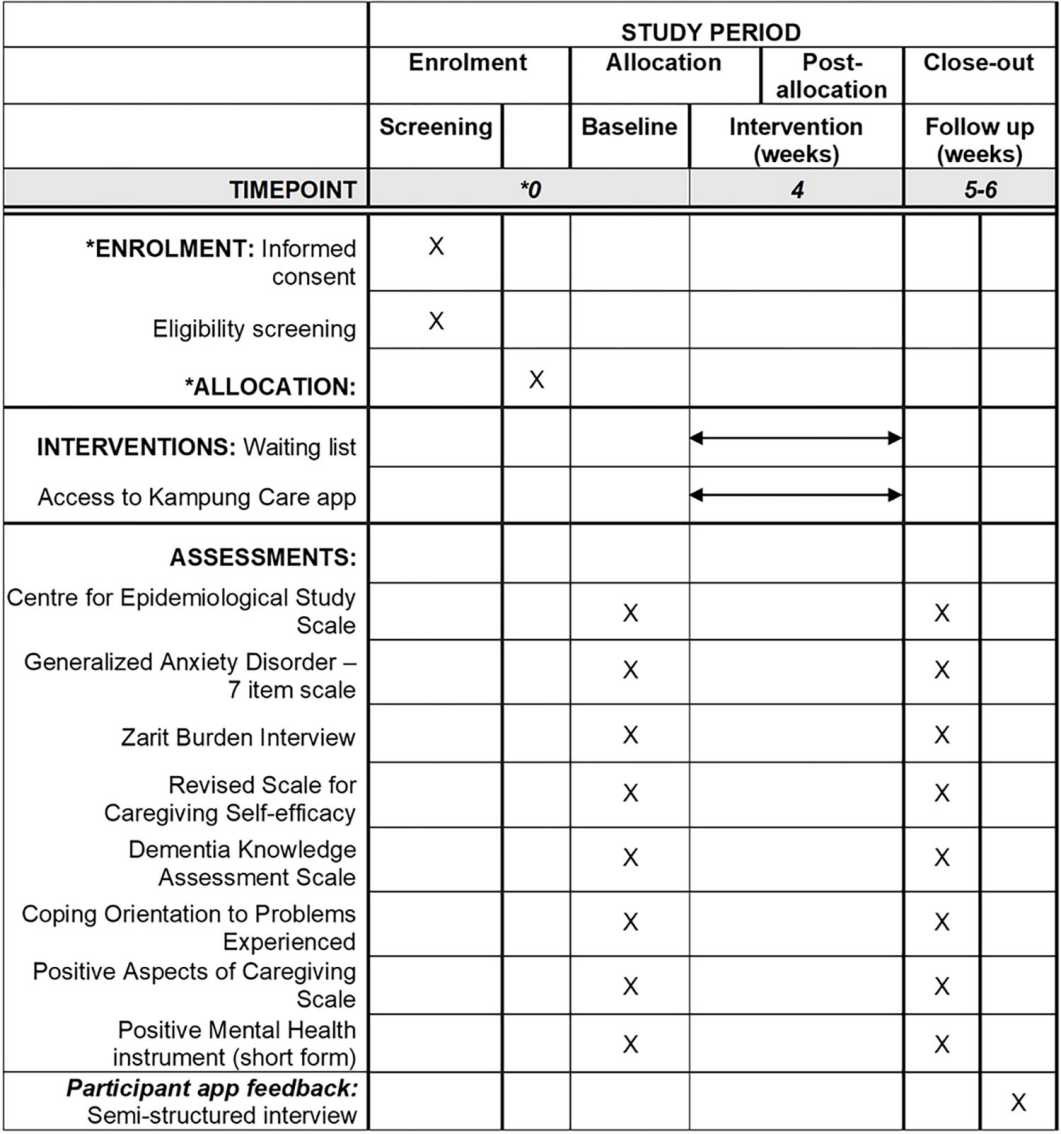
SPIRIT figure; schedule of enrolment, intervention, and assessments. *During enrolment, informed consent and eligibility screening will be conducted. Group allocation will be conducted immediately after if the participant meets the screening criteria. Thereafter, baseline assessments will be conducted immediately in the same session as well.

A study team member will assign participants to either the intervention or wait-list control group using block randomisation with a block size of four. An independent statistician not involved in the day-to-day administration of the project will conduct the randomisation by generating a sequence of numbers using the Sealed Envelope online tool on the randomisation website [41]. Allocation concealment is achieved by the ’sealed envelope’ method, in which the envelope contains a random code that must be checked against the randomisation list to determine group information. This is to prevent potential selection bias. Thereafter, the participant will be allocated into a group and complete the baseline assessment. One month later, a study team member will contact the participant to complete the post-assessment. Intervention group participants will also be invited to join a semi-structured interview to share their experience with the app and feedback on areas for improvement. Participants will be disbursed an inconvenience fee through cash or cashless modes of payment on completion of the baseline assessment (SGD25), post-assessment (SGD35), and follow-up interview (SGD30). Please refer to Fig 1 for the schedule of enrolment, intervention, and assessments. The approved study protocol can be found in S2 File.

**Fig 2:**
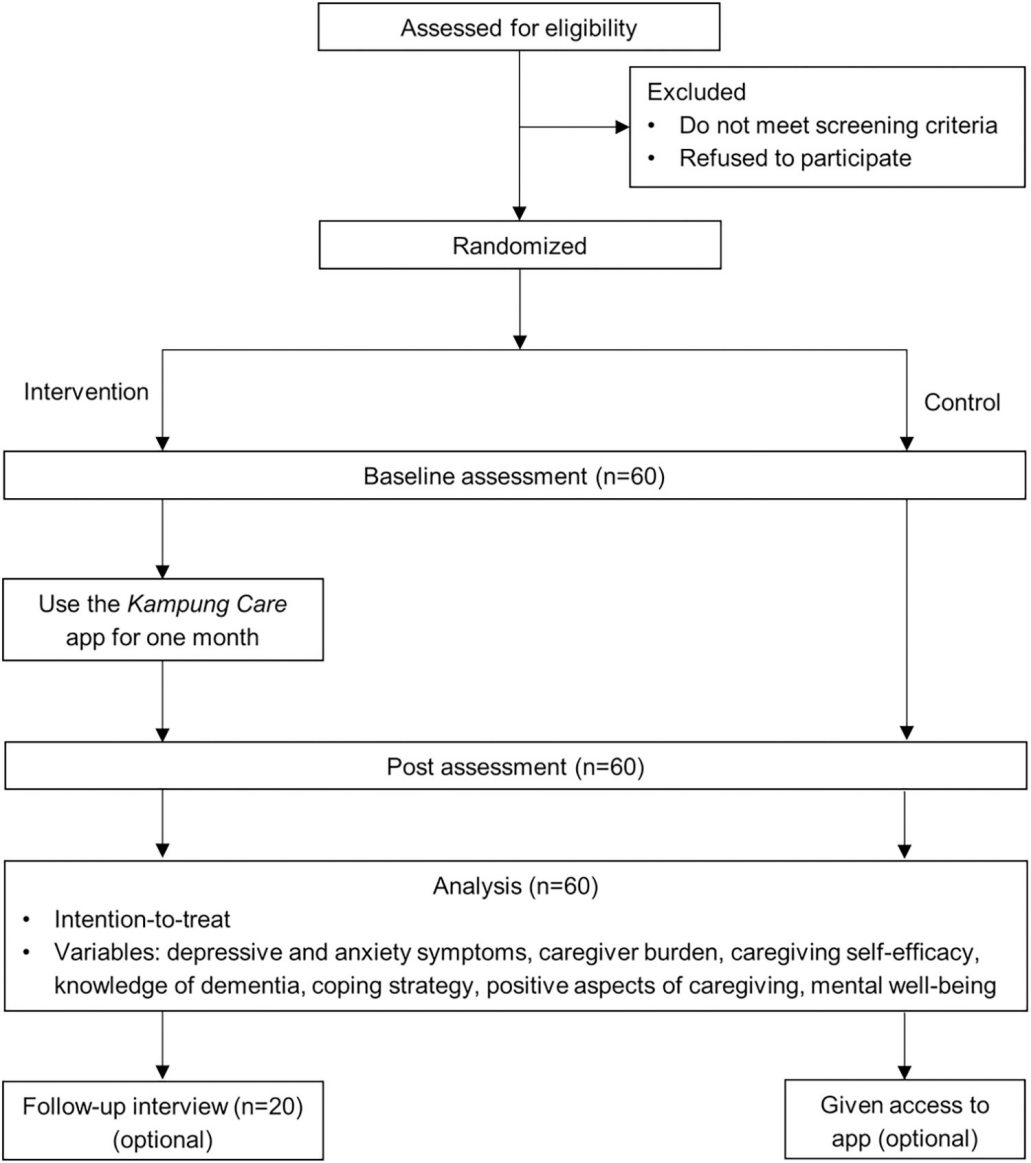
Flow chart of study procedure.

This study will be conducted in accordance with the latest version of the Declaration of Helsinki. Ethical approval for the study protocol was obtained from the National Healthcare Group’s Domain Specific Review Board (DSRB No: 2022/00029) (see S3 and S4 Files). Participation is voluntary and withdrawal from the study is possible at any point in time. Participants may also be asked to stop their participation at any time if they do not follow instructions required to complete the study adequately. All participants will provide written informed consent prior to their participation. Please refer to Fig 2 for the flow of the study procedure.

### Intervention components

**Fig 3:**
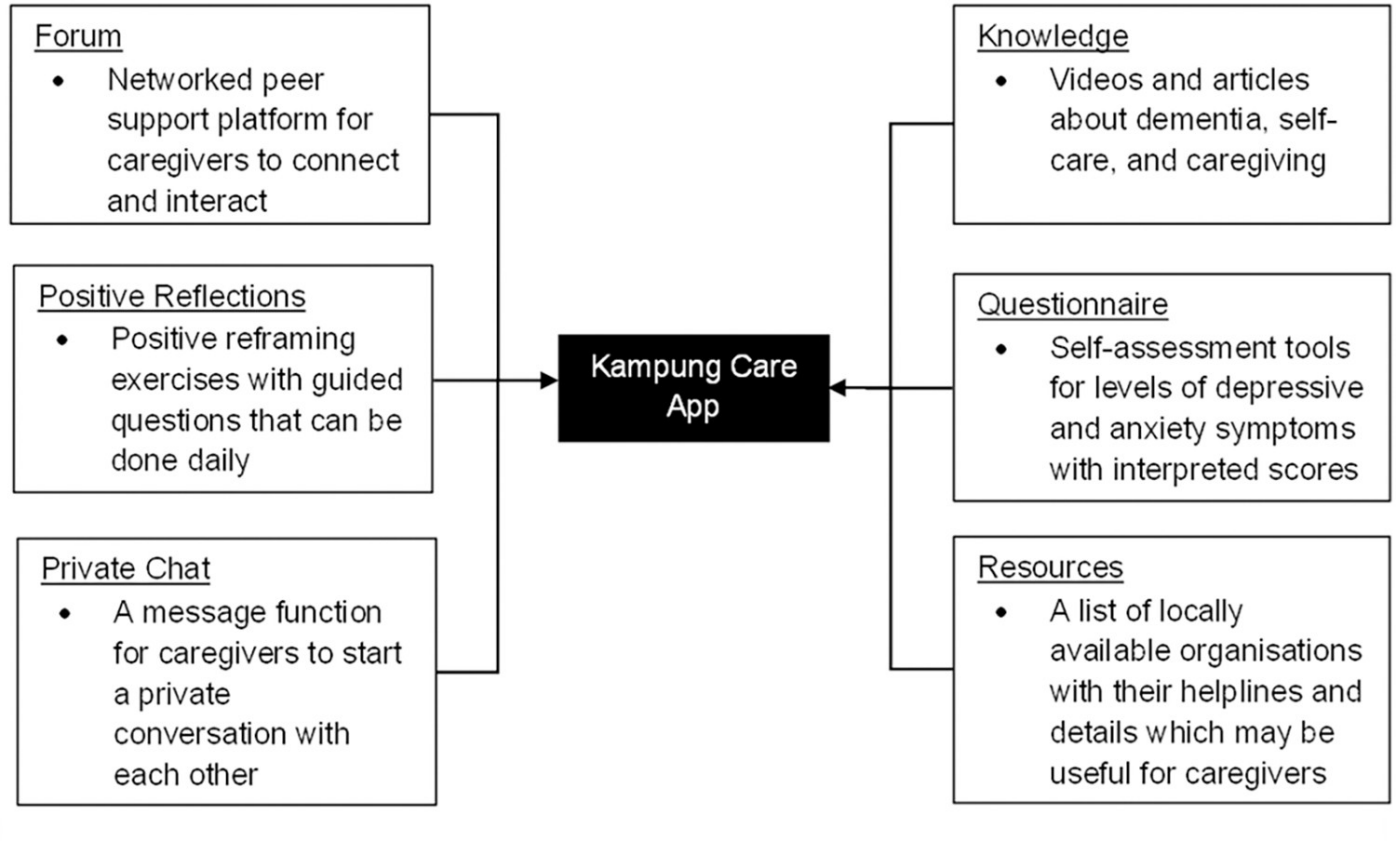
Summary of the Kampung Care app functions.

*Kampung Care* is a mobile app developed by the study team to promote the mental health of informal caregivers of PWD in Singapore. The app’s functions include a positive reflection journal, online peer support forum, private chat, knowledge base, self-assessment tools, and a list of locally available resources (see Fig 3).

These components were proposed based on the Stress and Coping model [7] and our previous studies amongst local informal caregivers of PWD [25, 29, 42–45]. Based on the Stress and Coping model [7], different coping strategies may lead to different health outcomes by affecting the stress appraisal process. By adopting a variety of coping strategies frequently, caregivers may improve their threshold in appraising an event as stressful [25]. The ‘positive reflections’ function was adapted from benefit-finding interventions in the literature [46, 47], which involve writing about one’s positive thoughts and feelings about a stressful experience. The intervention aims to impart positive reframing as an adaptive coping skill which caregivers can routinely utilise as a coping resource to deal with the stress from caregiving. Positive reframing is known to be associated with enhanced psychological outcomes, including lower psychological distress in family caregivers [48]. Furthermore, since caregivers spend much of their time on caring, having a self-assessment tool for them to gauge their depressive and anxiety levels (i.e., common problems which dementia caregivers face) can be a fast and cost-effective way to monitor their mental health and alert them of the need to engage in self-care practices if they are experiencing distress.

Additionally, local caregivers of PWD from our previous research perceived educational content (i.e., videos and knowledge related to dementia, caregiving, and self-care) and social and emotional support (i.e., forum, support group, list of support resources and helplines) to be beneficial features of digital health interventions [29]. Drawing on these findings, we included the forum, private chat, resource list, and knowledge base as our app’s functions. The forum and private chat aim to provide an outlet for carers to share their thoughts and experiences and connect with other caregivers. Users can post content, reply to others, and receive notifications when new interactions occur. These interactions can provide natural triggers to maintain platform usage and may increase caregivers’ engagement with the app [49]. As caregivers also expressed often needing support in managing care for PWD, having the resource list function would direct them to relevant organisations and resources that could be useful. Finally, the knowledge base function was designed to provide curated information for the different stages of dementia and knowledge which can improve dementia literacy, coping, caregiving, and self-care [29].

### Intervention delivery

The intervention group will be required to use the Kampung Care app for one month. Caregivers will be asked to complete at least two positive reflection journal entries per week (i.e., one in the middle of the week and one at the end of the week) and encouraged to use other app features. However, the actual usage of the app is ultimately dependent on users based on their schedule and interests during the one month. Follow-up calls will be made by a study team member weekly to seek feedback, provide clarification and assistance if needed, and to remind and motivate caregivers to continue app usage. During the call in the second week of app usage, the Patient Health Questionnaire-9 (PHQ-9) will be used to assess users’ mental health to monitor their risk and provide self-help resources if necessary. In the meantime, caregivers in the control group will be placed on a waiting list for one month.

After the end of one month, the post-assessment will be conducted for both groups. Scores from the post-assessment will be compared with that for the baseline to determine the effectiveness of the app. Caregivers’ app usage will also be analysed to examine links between usage patterns and intervention effects. Participants in the control group will also be given access to the app after completing the post-assessment, but they can refuse if they do not want access.

### Outcomes

Assessments to examine caregiver outcomes will be conducted at baseline and one month after baseline (post-assessment) for both the intervention and control groups. These will be conducted in English either online via videoconferencing or face-to-face at a location preferred by the participants.

The English version of the following questionnaires will be used in our study:

- Centre for Epidemiological Studies-Depression (CES-D) Scale
- Generalized Anxiety Disorder– 7 item scale
- Zarit Burden Interview
- Revised Scale for Caregiving Self-efficacy
- Dementia Knowledge Assessment Scale
- Coping Orientation to Problems Experienced
- Positive Aspects of Caregiving Scale
- Positive Mental Health instrument (short form)

The 20-item Centre for Epidemiological Study Scale (CES-D) will be used to measure the primary outcome–depressive symptoms amongst caregivers [50]. The instrument comprises 20 items, each rated on a 4-point Likert scale from 0 (*rarely or none of the time*) to 3 (*most or almost all the time*). The total score ranges from 0 to 60, with a score of 16 or higher indicating a risk for clinical depression [51]. The CES-D is a widely used scale which has been validated in Singapore [52] and was found to have an internal reliability of 0.91 when used in our previous study [25]. It also demonstrated good validity and reliability in detecting caregiver depression in another local study amongst caregivers of PWD [53]. Please see Table 1 for details on the secondary outcome measures.

**Table 1:** Measurement of secondary outcomes in the study.

Outcome	Questionnaire	Description
Anxiety	Generalized Anxiety Disorder– 7 item scale (GAD-7)	Each item of the Generalized Anxiety Disorder– 7 item scale (GAD-7) [54] is scored on a 4-point Likert scale from 0 (not at all) to 3 (nearly every day), with scores ranging from 0 to 21. A score of 10 or higher has been recommended as a cut-off point for identifying cases of GAD, while cut points of 5, 10, and 15 can be interpreted as mild, moderate, and severe levels of anxiety respectively [54]. The GAD-7 has been validated amongst the general population in Singapore [55] and used on a sample of local primary caregivers with a high internal consistency of 0.89 [56].
Caregiver burden	Zarit Burden Interview	The 22-item ZBI [57] assesses impairments on the caregiver’s health, psychological well-being, finances, and social life as a result of caregiving [58]. Each item is rated on a 5-point Likert scale from 0 (never) to 4 (nearly always present). A total score is obtained from summing up all items, with higher scores reflecting higher levels of burden. The instrument was previously validated in a local study in which it showed strong internal consistency, test-retest reliability, and construct validity when used on a sample of family caregivers [59]. In our prior research, the internal reliability of the ZBI was found to be 0.92 [25].
Caregivers’ level of self-efficacy	Revised Scale for Caregiving Self-efficacy (RSCSE)	The Revised Scale for Caregiving Self-efficacy (RSCSE) [60] comprises 15 items describing situations which caregivers may encounter regarding getting respite, responding to the care recipient’s memory and behavioral problems, and negative thoughts about their role as a caregiver. Caregivers will be asked to rate their level of confidence in overcoming these difficulties from 0 to 100, or not applicable if they feel that the item is not relevant to their current caregiving experience. The RSCSE is widely used globally and has been validated [61]. In our earlier study, the RSCSE’s 3-factor model was found to be robust and showed an acceptable fit amongst local caregivers [43].
Knowledge of dementia	Dementia Knowledge Assessment Scale (DKAS)	The 27-item Dementia Knowledge Assessment Scale (DKAS) [62] consists of statements on dementia, with some being factual, all with the response options: ‘*false*’, ‘*probably false*’, ‘*true*’, and ‘*probably true*’. For each item containing factual statements (e.g., ‘Exercise is generally beneficial for people experiencing dementia’), participants are given 1 or 2 points if they responded with ‘*probably true*’ or ‘*true*’ respectively, while all other responses are scored 0. For items with non-factual statements (e.g., ‘It is impossible to communicate with a person who has advanced dementia’), participants will be accorded 1 or 2 points if they responded with ‘*probable false*’ or ‘*false*’, respectively, while all other responses are accorded 0. The DKAS has been used in our previous studies amongst local caregivers of PWD [43, 63].
Coping strategy	brief Coping Orientation to Problems Experienced (COPE)	The brief Coping Orientation to Problems Experienced (COPE) [64] has 28 items measuring 14 different domains of coping, with two items for each domain. Caregivers are asked to rate how often they used each coping strategy to deal with stressful caregiving events during the past month on a 4-point Likert scale from 1 (‘*I haven’t been doing this at all’*) to 4 (‘*I’ve been doing this a lot’*) (Carver, 1997). This scale has been validated in a sample of caregivers of PWD, whereby it showed good internal consistency and construct validity [65]. When used in our earlier study, the brief COPE had an internal reliability of 0.88 [25]. It was also previously used amongst family caregivers of PWD in another local study [66].
Positive aspects of caregiving	Positive Aspects of Caregiving (PAC) Scale	Positive aspects of caregiving will be measured by the 9-item Positive Aspects of Caregiving (PAC) Scale [67], which is scored on a 5-point Likert scale ranging from 1 (*disagree a lot*) *to 5* (*agree a lot*). The overall PAC score ranges from 9 to 45, with a higher score reflecting a more positive perception of the caregiving experience. The reliability and validity of the PAC were established as acceptable amongst a sample of caregivers of older Singaporean adults with functional limitations [68], while other studies have also found similar results amongst caregivers of PWD [69, 70]. In our earlier study, the 2-factor structure of the PAC was found to be suitable when used amongst informal caregivers of PWD in Singapore [42].
Mental well-being	short Positive Mental Health (PMH) instrument	The short Positive Mental Health (PMH) instrument [71] is a short version of the original scale developed and validated in Singapore to measure the level of mental health in a general population [72]. It consists of 24 items, out of which 19 items assess six domains of mental health including general coping, emotional support, spirituality, interpersonal skills, personal growth and autonomy and global affect. Another five items are negatively worded filler items placed randomly to check for patterned responses. The ‘global affect’ subscale consists of three affect indicators which requires participants to rate from 1 (*never or very rarely*) to 5 (*very often or always*) how often they felt that over the past four weeks. For the rest of the subscales, participants are asked to indicate how much each item described them over the past 4 weeks on a scale from 1 (*not at all like me*) to 6 (*exactly like me*).

### Data analysis

The primary analysis will compare the outcome measures between the intervention and control groups by following the intention-to-treat principle. Additionally, per-protocol analyses will also be conducted. All analyses will be done through SPSS V29 or SAS 9.4. A two-sided p-value below 0.05 will be deemed statistically significant. Results will be reported according to the Consolidated Standards of Reporting Trials (CONSORT) statement regarding eHealth [73]. The statistician in charge of the final analysis will be blinded to group allocation to minimise bias. Repeated measures ANOVA analyses will be done to evaluate differences between the two groups over time. In the case of multiple comparisons, Bonferroni adjustment of p-values will be applied to avoid over-interpretation of the results. Effect size (i.e., Cohen’s d) will also be calculated. For the qualitative data, the interviews will be audio-recorded and transcribed before the analysis. Data analysis will be conducted using thematic analysis as informed by Braun and Clarke [74], where key themes will be identified and refined. A codebook will also be developed to serve as a coding framework for coding of the transcripts. Data management and qualitative analysis will be done through NVivo V11.

## Discussion

To the best of our knowledge, this study is the first in Singapore which will assess the feasibility and effectiveness of an app-based intervention for the mental health of informal caregivers of PWD. Despite a prevalent culture of digital usage and positive sentiments towards digital technologies in Singapore [28], mobile-based interventions to support dementia caregivers in the local setting remain scarce, not to mention ones that can target the multifaceted needs of caregivers of PWD. To maximise the feasibility and effectiveness of the app for caregivers, we drew on findings from our past studies [25, 29, 42–45] to ensure a user-centered app designed to address the experiences and needs of local caregivers. Our study can provide valuable insights which will contribute to the evidence base for the effectiveness of apps like *Kampung Care* in promoting the mental well-being of caregivers of PWD in Singapore.

Nevertheless, we foresee a few limitations of this study. The use of convenience sampling may render potential biases in the dataset, which calls for caution in over-generalising the results. Moreover, this study requires participants to be adept at using mobile applications, which may be a barrier for seniors or individuals who are less digitally literate to participate. As a result, the sample may not be representative of the mainstream population of informal caregivers of PWD. Another possible limitation of this study is a high dropout rate, but strategies to overcome this include weekly reminders to motivate participants to use the app and offering an inconvenience fee after each assessment as an incentive. Notwithstanding these limitations, the proposed *Kampung Care* app is an innovative intervention which could inform and encourage the future design and evaluation of app-based interventions to the mental well-being of local dementia caregivers.

### Trial status

Recruitment of participants commenced in December 2022 and will be open until the end of March 2024.

## Supporting information

S1 File SPIRIT 2013 checklist: Recommended items to address in a clinical trial protocol and related documents*. (DOCX)

S2 File Approved study protocol in ethics application form. (PDF)

S3 File Ethics approval. (PDF)

S4 File Extended ethics approval. (PDF)

## References

[1] Subramaniam M, Chong SA, Vaingankar JA, Abdin E, Chua BY, Chua HC, et al. Prevalence of Dementia in People Aged 60 Years and Above: Results from the WiSE Study. J Alzheimers Dis. 2015 Jan 1;45(4):1127–38. doi: 10.3233/JAD-142769 25672767

[2] Thompson JP, Riley CM, Eberlein RL. Modelling for insight: The case of dementia in Singapore. Syst Res Behav Sci. 2014 Mar;31(2):227–35.

[3] Woo LL, Thompson CL, Dong YH. Net informal costs of dementia in Singapore. J Clin Gerontol Geriatr. 2017;8(3):98–101.

[4] Brodaty H, Donkin M. Family caregivers of people with dementia. Dialogues Clin Neurosci. 2009;11(2):217–28. doi: 10.31887/DCNS.2009.11.2/hbrodaty 19585957 PMC3181916

[5] Peterson K, Hahn H, Lee AJ, Madison CA, Atri A. In the Information Age, do dementia caregivers get the information they need? Semi-structured interviews to determine informal caregivers’ education needs, barriers, and preferences. BMC Geriatr. 2016 Dec;16(1):1–13. doi: 10.1186/s12877-016-0338-7 27662829 PMC5035467

[6] Wiegelmann H, Speller S, Verhaert LM, Schirra-Weirich L, Wolf-Ostermann K. Psychosocial interventions to support the mental health of informal caregivers of persons living with dementia–a systematic literature review. BMC Geriatr. 2021;21(1):1–17. 33526012 10.1186/s12877-021-02020-4 PMC7849618

[7] Lazarus RS, Folkman S. Stress, appraisal, and coping. Springer publishing company; 1984.

[8] Hawken T, Turner-Cobb J, Barnett J. Coping and adjustment in caregivers: A systematic review. Health Psychol Open. 2018 Nov;5(2):2055102918810659. doi: 10.1177/2055102918810659 30450216 PMC6236498

[9] Tremont G. Family caregiving in dementia. Med Health R I. 2011;94(2):36. 21456372 PMC3487163

[10] Lazarus RS, Folkman S. Cognitive theories of stress and the issue of circularity. In: Dynamics of stress: Physiological, psychological and social perspectives. Boston, MA: Springer US; 1986. p. 63–80.

[11] Sörensen S, Conwell Y. Issues in dementia caregiving: effects on mental and physical health, intervention strategies, and research needs. Am J Geriatr Psychiatry. 2011 Jun 1;19(6):491–6. doi: 10.1097/JGP.0b013e31821c0e6e 21502853 PMC3774150

[12] Connors MH, Seeher K, Teixeira‐Pinto A, Woodward M, Ames D, Brodaty H. Dementia and caregiver burden: a three‐year longitudinal study. Int J Geriatr Psychiatry. 2020;35(2):250–8. doi: 10.1002/gps.5244 31821606

[13] Collins R, Kishita N. Prevalence of depression and burden among informal care-givers of people with dementia: A meta-analysis. Ageing Soc. 2020;40(11):2355–92.

[14] Sallim AB, Sayampanathan AA, Cuttilan A, Ho R. Prevalence of Mental Health Disorders Among Caregivers of Patients With Alzheimer Disease. J Am Med Dir Assoc. 2015;16(12):1034–41. doi: 10.1016/j.jamda.2015.09.007 26593303

[15] Cheng Y, Wang Z, Yang T, Lv W, Huang H, Zhang Y. Factors influencing depression in primary caregivers of patients with dementia in China: A cross-sectional study. Geriatr Nur (Lond). 2021;42(3):734–9. 10.1016/j.gerinurse.2021.03.017 33857837

[16] Fong TK, Cheung T, Chan WC, Cheng CP. Depression, Anxiety and Stress on Caregivers of Persons with Dementia (CGPWD) in Hong Kong amid COVID-19 Pandemic. Int J Environ Res Public Health. 2021;19(1):184. doi: 10.3390/ijerph19010184 35010451 PMC8751129

[17] Bandura A. Social cognitive theory: An agentic perspective. Annu Rev Psychol. 2001;52:1–26. doi: 10.1146/annurev.psych.52.1.1 11148297

[18] Losada-Baltar A, Falzarano FB, Hancock DW, Márquez-González M, Pillemer K, Huertas-Domingo C, et al. Cross-National Analysis of the Associations Between Familism and Self-Efficacy in Family Caregivers of People With Dementia: Effects on Burden and Depression. J Aging Health. 2023 Aug 10;0(0). doi: 10.1177/08982643231193579 37585806 PMC10858290

[19] Park J, Tolea MI, Arcay V, Lopes Y, Galvin JE. Self-efficacy and social support for psychological well-being of family caregivers of care recipients with dementia with Lewy bodies, Parkinson’s disease dementia, or Alzheimer’s disease. Soc Work Ment Health. 2019;17(3):253–78.

[20] Gkioka M, Teichmann B, Moraitou D, Papagiannopoulos S, Tsolaki M. Effects of a person centered dementia training program in Greek hospital staff—Implementation and evaluation. Brain Sci. 2020;10(12):976. doi: 10.3390/brainsci10120976 33322754 PMC7763588

[21] Pearlin LI, Mullan JT, Semple SJ, Skaff MM. Caregiving and the stress process: An overview of concepts and their measures. The gerontologist. 1990;30(5):583–94. doi: 10.1093/geront/30.5.583 2276631

[22] Monteiro AMF, Santos RL, Kimura N, Baptista MAT, Dourado MCN. Coping strategies among caregivers of people with Alzheimer disease: a systematic review. Trends Psychiatry Psychother. 2018;40:258–68. doi: 10.1590/2237-6089-2017-0065 30304119

[23] Quinn C, Toms G. Influence of positive aspects of dementia caregiving on caregivers’ well-being: A systematic review. The Gerontologist. 2019;59(5):584–96. doi: 10.1093/geront/gny168 30597058

[24] Pot AM, Blom MM, Willemse BM. Acceptability of a guided self-help Internet intervention for family caregivers: mastery over dementia. Int Psychogeriatr. 2015;27(8):1343–54. doi: 10.1017/S1041610215000034 25648589

[25] Yuan Q, Wang P, Tan TH, Devi F, Poremski D, Magadi H, et al. Coping patterns among primary informal dementia caregivers in Singapore and its impact on caregivers—Implications of a latent class analysis. The Gerontologist. 2021;61(5):680–92. doi: 10.1093/geront/gnaa080 32592582 PMC8276612

[26] Fiordelli M, Diviani N, Schulz PJ. Mapping mHealth research: a decade of evolution. J Med Internet Res. 2013;15(5):95. doi: 10.2196/jmir.2430 23697600 PMC3668610

[27] Handel MJ. mHealth (mobile health)-using Apps for health and wellness. Explore N Y N. 2011;7(4):256–61. doi: 10.1016/j.explore.2011.04.011 21724160

[28] Infocomm Media Development. Singapore digital society report 2023. [Internet]. 2023 [cited 2024 Feb 1]. Available from: https://www.imda.gov.sg/-/media/imda/files/infocomm-media-landscape/research-and-statistics/singapore-digital-society-report/singapore-digital-society-report-2023.pdf

[29] Samari E, Yuan Q, Zhang Y, Jeyagurunathan A, Subramaniam M. Barriers to using eHealth/mHealth platforms and perceived beneficial eHealth/mHealth platform features among informal carers of persons living with dementia: a qualitative study. BMC Geriatr. 2024;24(1):1–12. 38184551 10.1186/s12877-023-04628-0 PMC10771641

[30] Teles S, Ferreira A, Seeher K, Fréel S, Paúl C. Online training and support program (iSupport) for informal dementia caregivers: protocol for an intervention study in Portugal. BMC Geriatr. 2020;20(1):10. doi: 10.1186/s12877-019-1364-z 31914936 PMC6950829

[31] Yousaf K, Mehmood Z, Saba T, Rehman A, Munshi AM, Alharbey R, et al. Mobile-health applications for the efficient delivery of health care facility to people with dementia (PwD) and support to their carers: a survey. 2019. 10.1155/2019/7151475 PMC6457307 31032361

[32] Hopwood J, Walker N, McDonagh L, Rait G, Walters K, Iliffe S, et al. Internet-Based Interventions Aimed at Supporting Family Caregivers of People With Dementia. Syst Rev J Med Internet Res. 2018;20(6):216. 10.2196/jmir.9548 PMC6019848 29895512

[33] Shin Y, Kim SK, Kim Y, Go Y. Effects of app-based mobile interventions for dementia family caregivers: a systematic review and meta-analysis. Dement Geriatr Cogn Disord. 2022;51(3):203–13. doi: 10.1159/000524780 35609526

[34] Zhao Y, Feng H, Hu M, Hu H, Li H, Ning H, et al. Web-Based Interventions to Improve Mental Health in Home Caregivers of People With Dementia: Meta-Analysis. J Med Internet Res. 2019;21(5):13415. 10.2196/13415 PMC6526687 31066680

[35] Castillo LI, Tran V, Hadjistavropoulos T. Are mobile apps meeting the needs of caregivers of people living with dementia? An evaluation of existing apps for caregivers. Aging & Mental Health; 2024 Apr;28(4):577–86. doi: 10.1080/13607863.2023.2177832 36775643

[36] Brown EL, Ruggiano N, Li J, Clarke PJ, Kay ES, Hristidis V. Smartphone-based health technologies for dementia care: opportunities, challenges, and current practices. J Appl Gerontol. 2019;38(1):73–91. doi: 10.1177/0733464817723088 28774215

[37] Rathnayake S, Moyle W, Jones C, Calleja P. mHealth applications as an educational and supportive resource for family carers of people with dementia: An integrative review. Dementia. 2019;18(7–8):3091–112. doi: 10.1177/1471301218768903 29631492

[38] Dementia Singapore. New Launch: CARA Mobile App. 2021 [cited 2024 Feb 1]. Available from: https://dementia.org.sg/2021/11/23/new-launch-cara-mobile-app/

[39] Whitehead AL, Julious SA, Cooper CL, Campbell MJ. Estimating the sample size for a pilot randomised trial to minimise the overall trial sample size for the external pilot and main trial for a continuous outcome variable. Stat Methods Med Res. 2016;25(3):1057–73. doi: 10.1177/0962280215588241 26092476 PMC4876429

[40] Bédard M, Molloy D, Squire L, Dubois S, Lever JA, O’Donnell M. The Zarit Burden Interview: a new short version and screening version. The gerontologist. 2001;41(5):652–7. doi: 10.1093/geront/41.5.652 11574710

[41] Create a blocked randomisation list | Sealed Envelope [Internet]. [cited 2024 Apr 26]. Available from: https://www.sealedenvelope.com/simple-randomiser/v1/lists

[42] Devi F, Yuan Q, Wang P, Tan GTH, Roshan Goveas R, Ng LL, et al. Positive aspect of caregiving among primary informal dementia caregivers in Singapore. PloS One. 2020;15(8):0237677. doi: 10.1371/journal.pone.0237677 32817648 PMC7440648

[43] Tan GTH, Yuan Q, Devi F, Wang P, Ng LL, Goveas R, et al. Factors associated with caregiving self-efficacy among primary informal caregivers of persons with dementia in Singapore. BMC Geriatr. 2021;21(1):1–11. 33407201 10.1186/s12877-020-01951-8 PMC7789728

[44] Yuan Q, Tan TH, Wang P, Devi F, Ong HL, Abdin E, et al. Staging dementia based on caregiver reported patient symptoms: Implications from a latent class analysis. PloS One. 2020;15(1):0227857. doi: 10.1371/journal.pone.0227857 31940419 PMC6961931

[45] Yuan Q, Tan GTH, Wang P, Devi F, Goveas R, Magadi H, et al. Combining a variable‐centered and a person-centered analytical approach to caregiving burden–a holistic approach. BMC Geriatr. 2021;21(1):1–11. 33931027 10.1186/s12877-021-02238-2 PMC8086073

[46] Cheng ST, Lau RW, Mak EP, Ng NS, Lam LC. Benefit-finding intervention for Alzheimer caregivers: conceptual framework, implementation issues, and preliminary efficacy. The Gerontologist. 2014 Dec 1;54(6):1049–58. doi: 10.1093/geront/gnu018 24688081

[47] Brand C, O’Connell BH, Gallagher S. A randomised controlled trial of benefit finding in caregivers: The Building Resources in Caregivers Study Protocol. Health Psychol Open. 2015 Jul 9;2(2). doi: 10.1177/2055102915595019 28070362 PMC5193262

[48] Del-Pino-Casado R, Serrano-Ortega N, López-Martínez C, Orgeta V. Coping strategies and psychological distress in family carers of frail older people: A longitudinal study. J Affect Disord. 2019 Sep 1;256:517–23. doi: 10.1016/j.jad.2019.06.038 31280075

[49] Fogg BJ. Creating persuasive technologies: an eight-step design process. In: Proceedings of the 4th international conference on persuasive technology. 2009. p. 1–6.

[50] Radloff LS. The CES-D scale: A self-report depression scale for research in the general population. Appl Psychol Meas. 1977;1(3):385–401.

[51] Lewinsohn PM, Seeley JR, Roberts RE, Allen NB. Center for Epidemiologic Studies Depression Scale (CES-D) as a screening instrument for depression among community-residing older adults. Psychol Aging. 1997;12(2):277. doi: 10.1037//0882-7974.12.2.277 9189988

[52] Stahl D, Sum CF, Lum SS, Liow PH, Chan YH, Verma S, et al. Screening for depressive symptoms: validation of the center for epidemiologic studies depression scale (CES-D) in a multiethnic group of patients with diabetes in Singapore. Diabetes Care. 2008;31(6):1118–9. doi: 10.2337/dc07-2019 18337303

[53] Ying J, Yap P, Gandhi M, Liew TM. Validity and utility of the Center for Epidemiological Studies Depression Scale for detecting depression in family caregivers of persons with dementia. Dement Geriatr Cogn Disord. 2019;47(4–6):323–34. doi: 10.1159/000500940 31307034 PMC6878745

[54] Spitzer RL, Kroenke K, Williams JB, Löwe B. A brief measure for assessing generalized anxiety disorder: the GAD-7. Arch Intern Med. 2006;166(10):1092–7. doi: 10.1001/archinte.166.10.1092 16717171

[55] Lim L, Ng TP, Chua HC, Chiam PC, Won V, Lee T, et al. Generalised anxiety disorder in Singapore: prevalence, co-morbidity and risk factors in a multi-ethnic population. Soc Psychiatry Psychiatr Epidemiol. 2005;40:972–9. doi: 10.1007/s00127-005-0978-y 16249971

[56] Jeyagurunathan A, Sagayadevan V, Abdin E, Zhang Y, Chang S, Shafie S, et al. Psychological status and quality of life among primary caregivers of individuals with mental illness: a hospital based study. Health Qual Life Outcomes. 2017;15:1–14. 28526049 10.1186/s12955-017-0676-y PMC5438522

[57] Zarit SH, Reever KE, Bach-Peterson J. Relatives of the impaired elderly: correlates of feelings of burden. The gerontologist. 1980;20(6):649–55. doi: 10.1093/geront/20.6.649 7203086

[58] Lau JH, Abdin E, Jeyagurunathan A, Seow E, Ng LL, Vaingankar JA, et al. The association between caregiver burden, distress, psychiatric morbidity and healthcare utilization among persons with dementia in Singapore. BMC Geriatr. 2021;21:1–10. 33468059 10.1186/s12877-021-02014-2 PMC7816438

[59] Seng BK, Luo N, Ng WY, Lim J, Chionh HL, Goh J, et al. Validity and reliability of the Zarit Burden Interview in assessing caregiving burden. Ann Acad Med Singapore. 2010;39(10):758–63. 21063635

[60] Steffen AM, McKibbin C, Zeiss AM, Gallagher-Thompson D, Bandura A. The revised scale for caregiving self-efficacy: reliability and validity studies. J Gerontol B Psychol Sci Soc Sci. 2002;57(1):74–86. doi: 10.1093/geronb/57.1.p74 11773226

[61] Steffen AM, Gallagher-Thompson D, Arenella KM, Au A, Cheng ST, Crespo M, et al. Validating the revised scale for caregiving self-efficacy: a cross-national review. The Gerontologist. 2019;59(4):325–42. doi: 10.1093/geront/gny004 29546334

[62] Annear MJ, Toye CM, Eccleston CE, McInerney FJ, Elliott KEJ, Tranter BK, et al. Dementia knowledge assessment scale: development and preliminary psychometric properties. J Am Geriatr Soc. 2015;63(11):2375–81. doi: 10.1111/jgs.13707 26503020

[63] Tan GTH, Yuan Q, Devi F, Wang P, Ng LL, Goveas R, et al. Dementia knowledge and its demographic correlates amongst informal dementia caregivers in Singapore. Aging Ment Healt. 2021;25(5):864–72. doi: 10.1080/13607863.2020.1740914 32228179

[64] Carver CS. You want to measure coping but your protocol’too long: Consider the brief cope. Int J Behav Med. 1997;4(1):92–100. 16250744 10.1207/s15327558ijbm0401_6

[65] Cooper C, Katona C, Livingston G. Validity and reliability of the brief COPE in carers of people with dementia: the LASER-AD Study. J Nerv Ment Dis. 2008;196(11):838–43. doi: 10.1097/NMD.0b013e31818b504c 19008735

[66] Lim J, Griva K, Goh J, Chionh HL, Yap P. Coping strategies influence caregiver outcomes among Asian family caregivers of persons with dementia in Singapore. Alzheimer Dis Assoc Disord. 2011;25(1):34–41. doi: 10.1097/WAD.0b013e3181ec18ae 20693866

[67] Tarlow BJ, Wisniewski SR, Belle SH, Rubert M, Ory MG, Gallagher-Thompson D. Positive aspects of caregiving: Contributions of the REACH project to the development of new measures for Alzheimer’s caregiving. Res Aging. 2004;26(4):429–53.

[68] Siow JYM, Chan A, Østbye T, Cheng GHL, Malhotra R. Validity and reliability of the positive aspects of caregiving (PAC) scale and development of its shorter version (S-PAC) among family caregivers of older adults. The Gerontologist. 2017;57(4):75–84. doi: 10.1093/geront/gnw198 28082275

[69] Abdollahpour I, Nedjat S, Noroozian M, Salimi Y, Majdzadeh R. Positive aspects of caregiving questionnaire: a validation study in caregivers of patients with dementia. J Geriatr Psychiatry Neurol. 2017;30(2):77–83. 28077010 10.1177/0891988716686831

[70] Grover S, Nehra R, Malhotra R, Kate N. Positive aspects of caregiving experience among caregivers of patients with dementia. East Asian Arch Psychiatry. 2017;27(2):71–8. 28652500

[71] Vaingankar JA, Subramaniam M, Abdin E, Picco L, Chua BY, Eng GK, et al. Development, validity and reliability of the short multidimensional positive mental health instrument. Qual Life Res. 2014;23:1459–77. doi: 10.1007/s11136-013-0589-0 24307210

[72] Vaingankar JA, Subramaniam M, Chong SA, Abdin E, Orlando Edelen M, Picco L, et al. The positive mental health instrument: development and validation of a culturally relevant scale in a multi-ethnic Asian population. Health Qual Life Outcomes. 2011;9:1–18. 22040157 10.1186/1477-7525-9-92 PMC3229450

[73] Eysenbach G, Group CEHEALTH. CONSORT-EHEALTH: improving and standardizing evaluation reports of Web-based and mobile health interventions. J Med Internet Res. 2011;13(4):1923. doi: 10.2196/jmir.1923 22209829 PMC3278112

[74] Braun V, Clark V. What can “thematic analysis” offer health and wellbeing researchers?. Int J Qual Stud Health Well-Being. 2014 Jan 1;9(1):26152. 25326092 10.3402/qhw.v9.26152 PMC4201665

