# Supplementary material for: Evaluating a mobile-based intervention to promote the mental health of informal dementia caregivers in Singapore: Study protocol for a pilot two-armed randomised controlled trial
Source: PLoS One. 2024 Jun 24;19(6):e0305729. doi: 10.1371/journal.pone.0305729 (PMC11195940; doi:10.1371/journal.pone.0305729)
Supplement: S2 File — (PDF) [file pone.0305729.s002.pdf]

Study Reference Number: 2022/00029

Version Number: 2

I declare that the study falls under the purview of the Human Biomedical Research Act.*Click here for help

◉ Yes
○ No

Please tick all the boxes that apply:

My human biomedical research is intended to study -

☑ (a) the prevention, prognostication, diagnosis or alleviation of any disease, disorder or injury affecting the human body;
☐ (b) the restoration, maintenance or promotion of the aesthetic appearance of human individuals through clinical procedures or techniques; or
☐ (c) the performance or endurance of human individuals,

Where the research involves -

☑ (i) subjecting an individual to any intervention (including any wilful act or omission) that has a physical, mental or physiological effect (whether temporary or permanent) on the body of the individual;
☐ (ii) the use of any individually-identifiable human biological material; or
☐ (iii) the use of any individually-identifiable health information.

A1. Please enter the full study title.

Design and Pilot-test of an Innovative Mobile-based Intervention to Promote Mental Health of Informal Dementia Caregivers

A2. (Optional) Please assign Study Administrators below.* Click here for help

No.	Name	Institution	Department	Role	Email
1	Mythily Subramaniam	Institute of Mental Health	Research Division	Study Administrator	mythily@ imh.com.sg

B1. (a) (Optional) Overall Principal Investigator Click here for help: Please include the name, designation and institution. ____________________

B1. (b) Submitting Principal Investigator (Main Contact for DSRB): **Qi YUAN**

B2 Study Sites under the oversight of NHG DSRB (Please click here to view complete list)

No.	Study Site	Name	Study Role	Institution	Department	Min Training
1	Institute of Mental Health	Dr Qi YUAN	PI	Institute of Mental Health	Research Division	Completed
2	Institute of Mental Health	Dr Mythily Subramaniam	Co-Investigator	Institute of Mental Health	Research Division	Completed
3	Institute of Mental Health	Dr Edimansyah Abdin	Co-Investigator	Institute of Mental Health	Research Division	Completed
4	Institute of Mental Health	Ms ZHANG YUNJUE	Co-Investigator	Institute of Mental Health	Research Division	Completed
5	Institute of Mental Health	Ms Ellaisha Binte Samari	Co-Investigator	Institute of Mental Health	Research Division	Completed
6	Institute of Mental Health	Dr Richard Roshan Goveas	Co-Investigator	Institute of Mental Health	Geriatric Psychiatry	Completed
7	Institute of Mental Health	Dr NG LI LING	Co-Investigator	Changi General Hospital (CGH)	Psychological Medicine	Completed
8	Institute of Mental Health	Mr LIU LEI	Co-Investigator	Institute of Mental Health	Nursing	Biomedical CITI - Completed
9	Institute of Mental Health	Ms Yun Ting Lee	Co-Investigator	Institute of Mental Health	Research Division	Biomedical CITI - Completed<br>FCOI CITI - Completed

B3. External Study Sites under the supervision of the 'Submitting Principal Investigator (Main contact for DSRB)' (eg. Nursing Home, Community Hospitals, Community Centres etc). Please attach the Notice of Intent (NoI) if applicable. Click here for help

No.	Study Site	Institution Authorisation	Engaged in research	IRB Approval	Contact Person
There is nothing.					

B4 External Study Site (for Institutions NOT under the oversight of NHG DSRB)

(a) to another IRB?

◉ No
○ Yes, please state the IRB.

(b) Has the application been previously rejected by any IRB? (Including NHG-DSRB)

◉ No
○ Yes

B5 Research Specialty

(a) Please indicate the Special / Research Expertise*

No.	Primary Specialty	Primary Sub Specialty	Others
1	Public Health	Others	mental health

(b) Please indicate/add Secondary Special / Research Expertise

No.	Primary Specialty
There is nothing.	

With effect from 1 January 2015, all study team members involved in the design, conduct or reporting of the research are required to complete and endorse a Conflict of Interest Declaration Form annually to the DSRB Financial Conflict of Interest (FCOI) Secretariat. This declaration includes any conflicts of interest of their immediate family members (includes parents, siblings, spouse and each dependent child).

The annual Conflict of Interest Declaration Cycle will be from 01 Jan to 31 Jan of the year and the declaration will be valid from 1 Jan to 31 Dec of the same year. The Conflict of Interest Declaration Form may still be submitted beyond the Declaration Cycle. However, the declaration will only be valid until the next Declaration Cycle.The Conflict of Interest Declaration Form can be downloaded from https://www.research.nhg.com.sg/wps/wcm/connect/romp/nhgromp/hspp/financial+conflict+of+interest/fcoi+policy.

An updated Conflict of Interest Declaration Form must be submitted to the FCOI Secretariat as soon as possible but no later than 30 days if any of the circumstances relevant described herein change during the conduct of the research.

Dr Qi YUAN (Principal Investigator)
- ○ Yes
- ◉ No

Dr Mythily Subramaniam (Co-Investigator)
- ○ Yes
- ◉ No

Dr Edimansyah Abdin (Co-Investigator)
- ○ Yes
- ◉ No

Ms ZHANG YUNJUE (Co-Investigator)
- ○ Yes
- ◉ No

Ms Ellaisha Binte Samari (Co-Investigator)
- ○ Yes
- ◉ No

Dr Richard Roshan Goveas (Co-Investigator)
- ○ Yes
- ◉ No

Dr NG LI LING (Co-Investigator)
- ○ Yes
- ◉ No

Mr LIU LEI (Co-Investigator)
- ○ Yes
- ◉ No

Ms Yun Ting Lee (Co-Investigator)
- ○ Yes
- ◉ No

Please attach the Study Team Member List if there are any study team members (study coordinators, biostatisticians etc.) involved in the design, conduct and reporting of the research, who are not listed in Section B and C of the DSRB Application Form.

D1 Please select the Nature(s) of Study that best describes your application

☐ Education Research
☐ Health Services and Outcome Research
☑ Prevention & Health Promotion Programme
☐ Epidemiological Research
☐ Social and Behavioral Research
☐ Community-based Participatory Research
☐ Others

D2 Please tick all research method(s) that is/are relevant to your study:* Click here for help

☑ Questionnaire or Survey
☐ Analysis of Existing Data
☑ Focus Group Discussion / Interview
☐ Clinical Research

E1. Who will be responsible for the payment and compensation of injury or illness arising from participation of research participants in the study?

- ◉ National Clinical Trial (CT) Insurance Policy (All public healthcare institutions are covered under this policy. You may contact your institution research office for more information if required.)
- ○ Sponsor
- ○ Others

E2 Please give information regarding the study's Funding source or Sponsor information.

- ○ No funding is required for this study to be carried out
- ○ Pharmaceutical / Industry Sponsored
- ◉ Grant/ other source(s) of funding

i. Name of Grant Agency and Grant Name (For other source(s) of funding, please select 'Others' and proceed to specify the funding source(s) in the next text box) Others

Please specify: NHG Population Health Grant

ii. Grant/ funds amount applied for 183,919

iii. Date of Grant/ funding application deadline (For other source(s) of funding, please enter the effective date of the contract) 01-Mar-2021

iv.Has the Grant/ funding application been approved?

◉ Yes. Grant/ funding application successful.

Date of Grant/ funding Approval: 03-Nov-2020

Date of Grant/ funding Expiry: 01-Jun-2023

Amount of Grant/ funding awarded: 183,919

Please attach the grant approval letter or notification of award. However, if your study is a US federally funded research, please attach the approved grant proposal and all relevant documents approved by the grant body (e.g. study protocol, consent form etc.).

LOA_App for Dementia Caregivers.pdf

Approval of grant extension 1 Jun 2023 _ PHG project (PHG20.P.I.2.6) Design and pilot-test of an innovative mobile-based intervention.pdf

○ No. Grant/ funding application is pending approval.

○ Financing/Sponsorship from Community Based Agency

◉ Non- Exempt (Expedited / Full Board)
○ Exempt. Please note that if your study does not qualify as exempt, the application for Non-Exempt review must be completed.Please click on "Exempt" to find out more on the list

G1 What are the specific aims of this study?

The current project aims to 1) 1) design and develop a mobile-based multi-component intervention (i.e. an app) to promote mental health among informal caregivers of individuals with dementia in Singapore; 2) test the effectiveness of a mobile-based intervention (i.e. App) on promoting the mental health of informal caregivers of persons with dementia in Singapore through a pilot randomized control trial; 2) collect users' feedback on the intervention for its future improvements.

G2 What is the Hypothesis of this study? For qualitative studies, please provide the research question instead.

We hypothesize that the intervention (i.e. the App) would lower the reported depressive symptoms among local dementia caregivers. It will also improve their knowledge of dementia, caregiving efficacy, positive coping strategy, perceived positive aspects of caregiving, and their mental well-being; and reduce their caregiving burden, and level of anxiety, compared to the control group.

G3 Please describe the background to the current study proposal. Critically evaluate the existing knowledge and specifically identify the gaps that the proposed study is intended to fill.

The prevalence of dementia was about 10% among residents aged 60 years and above in Singapore according to a national study conducted by the Institute of Mental Health in 2013 (Subramaniam, Chong et al. 2015), which was equivalent to 51,934 older adults locally (WiSE team and Institute of Mental Health Research Division 2014). One significant concern for persons with dementia (PWD) is that they usually show high dependence and require intensive care, mostly from informal caregivers such as family members (Schulz and Martire 2004, Brodaty and Donkin 2009, Haro, Kahle-Wrobleski et al. 2014). The global financial cost associated with dementia was reported as US$ 948 billion in 2016, among which 42.3% were from informal care (Xu, Zhang et al. 2017). Similarly, the estimated societal costs of dementia in Singapore had reached SGD 532 million in 2013 (Abdin, Subramaniam et al. 2016). Since the population in Singapore is aging, this amount is expected to increase as well. Although caregiving could be rewarding (Kramer 1997, Cohen, Colantonio et al. 2002), family members caring for PWD at home often describe the experience as enduring, stressful, and frustrating (Butcher, Holkup et al. 2001), making them one of the high-risk groups for potential health issues. Previous research had identified several negative consequences of caregiving, such as caregiver burden (Haro, Kahle-Wrobleski et al. 2014), depressive symptoms (Givens, Mezzacappa et al. 2014), and poor self-perceived physical health (Pinquart and Sörensen 2007). According to a previous systematic review, the aggregate prevalence of depression was reported to be 34% in a previous meta-analysis (Sallim, Sayampanathan et al. 2015). Once the informal caregiver develops a mental illness, the quality of care received by PWD would deteriorate and they might be institutionalized more quickly (Brodaty and Donkin 2009). This in turn will increase the financial burden of dementia. To address the above concerns and also to improve the life quality of PWD, promoting the mental health of dementia caregivers could be one essential endeavor.Due to their heavy involvement in daily caregiving, caregivers usually have difficulties in attending face-to-face interventions. For example, a survey of 227 US dementia caregivers suggested that nearly a quarter of them provided 40 hours of care or more per week (Alzheimer's Association and National Alliance for Caregiving 2004). Another study among primary caregivers in Greece (n=161) reported a mean weekly caregiving time of more than 70 hours (Mougias, Politis et al. 2015). This number is similarly striking among primary informal caregivers in Singapore. According to our recent study among local informal caregivers of PWDs, the average weekly hours spent on caregiving were about 55 hours (Yuan, Wang et al. 2020). And this situation might be even worse in the current COVID-19 outbreak. To better support them now and in the future, an alternative could be to rely on a mobile-based intervention, as the penetration rate of smartphones among local residents is quite high (aged 15-49: >95%, aged 50-59: 88%, and aged 60 and above: 56%) (Infocomm Media Development Authority of Singapore 2018). Mobile apps have been widely used in supporting caregivers of older adults - a review identified 44 apps as of Oct 2017, and findings suggested that it’s a viable way to reduce the burden and improve the health outcomes of caregivers (Grossman, Zak et al. 2018). For caregivers of PWDs, although the number of apps is relatively smaller, preliminary studies suggested the feasibility and acceptance of this method (Reyes, Camargo et al. 2015, Brown, Ruggiano et al. 2016, Callan, Siegle et al. 2016, Rathnayake, Moyle et al. 2018). However, reviews of mobile apps for dementia caregivers did identify a few gaps. For example, in 2017 Brown et al. (Brown, Ruggiano et al. 2017) identified 13 mobile apps in their review (all developed in western countries), and only 5 of them had a caregiver focus; and

individual apps only had limited functions which could not meet the complex need of caregivers, suggesting the need of multi-component programs. Another review in 2018 identified 6 different mobile apps to support dementia caregivers and had been used in different areas including education, monitoring, and cognitive training; however, it also found that there is a lack of user-centered design in app development and rigorously designed studies based on a clear theoretical framework (Rathnayake, Moyle et al. 2018). More importantly, none of these Apps identified in these two reviews were Singapore-based (Brown, Ruggiano et al. 2017, Rathnayake, Moyle et al. 2018). To the best of our knowledge, there is one mobile app that used to be available for dementia caregivers in Singapore (i.e. eCareApp). However, this app ceased to function since mid-Aug 2018. There is another app 'DementiaFriends' available in the market, aiming to help caregivers to find PWD if they get lost. The research gap still exists in terms of how to better support local caregivers of PWD, to provide them with the helpful cultural relevant knowledge, support, and resources across different stages of PWD, to help both the caregiver and the patient to get well prepared through this process and to improve perceived burden and health outcomes of caregivers. Furthermore, evidence is still needed in terms of the effectiveness of such mobile-based supporting programs among local informal caregivers. This is the first study in Singapore that proposes to promote the mental health of informal caregivers of PWDs through a mobile-based intervention. We will work with a third-party vendor to develop the App. The main components of the App include dementia knowledge, a networked peer support platform, positive reflection, and information on locally available resources. These components were proposed based on our previous research studies among local informal caregivers of PWD (Devi, Yuan et al. 2020, Yuan, Tan et al. 2020, Yuan, Wang et al. 2020, Tan, Yuan et al. 2021, Yuan, Tan et al. 2021), and the 'Stress and coping model' (Lazarus and Folkman 1984). The current study will serve as a reference for the development and evaluation of future evidence-based m-health interventions in the local setting, and it might stimulate more research in relevant areas.

G4 Please provide details on (i) sample size and power calculation and (ii) the means by which data will be analyzed and interpreted. If this is a pilot study/qualitative study and no sample size calculation is performed, please provide a rationale on how the recruitment target is determined.

During the app design and development phase, focus group discussions will be conducted to collect feedback from potential users once the prototype of the app is ready. The planned sample size is 15, however, the final sample will be determined by data saturation. The group size will vary from 4-6 in considering their heavy involvement in the daily caregiving and the fact that we only have a short window period to collect such information (2- 4 weeks). Since the current study mainly aims to explore the feasibility of the mobile-based intervention and its potential effectiveness among local informal caregivers of PWDs, a pilot study with 100 participants is planned for the randomized control trial (RCT) phase. Block randomization with a block size of 4 will be used to allocate the caregivers into either the intervention (n=50) or the waiting-list control condition (n=50). The sample size for the current pilot was determined with the following rationales: according to a previous methodological study, a sample size of 25 per arm is required for the pilot if the main trial will be designed with 90% power and two-sided 5% significance for an invention with a small effect size (Whitehead, Julious et al. 2016). However, since mobile-based intervention studies usually have high attrition rates – for example, an overall attrition rate of 43% was reported for app-based interventions for chronic disease according to a previous systematic review (Meyerowitz-Katz, Ravi et al. 2020). As such, we have doubled the sample required from 25 to 50 to minimize the situation that there will be not enough samples for the analysis. After the RCT phase, participants in the intervention group will be invited to join a separate in-depth interview to collect their feedback and comments on the App and to explore how to improve the app in the future. We proposed 20 participants for this feedback phase. The final sample of this qualitative sub-study depends on data saturation. In other words, we will stop the recruitment if no new findings from the interviews even if we interview less than 20 participants. For the RCT, results will be reported following the Consolidated Standards of Reporting Trials (CONSORT) statement regarding eHealth (Eysenbach and Group 2011). Group information will not be disclosed to the statistician who is in charge of the final analysis, this is to blind the data analyst to reduce bias (Karanicolas, Farrokhyar et al. 2010). The primary analysis will follow the intention-to-treat principle and compare the outcome measures between the intervention vs. control groups. In addition, per-protocol analyses will also be conducted. More specifically, descriptive statistics will be used to describe the participants' profiles and their characteristics. The Mann–Whitney U-test will be used to assess any significant differences in the demographic data and clinical characteristics of the two groups. A multivariate repeated measured ANOVA will be performed to examine the differences between the two groups over time, with the adjustment of potential baseline differences. Effect size (i.e. Cohen's d) will

also be calculated. All analysis will be conducted through SPSS V21 or SAS 9.4.For the qualitative data, the discussion and interviews will be audio-recorded and transcribed before the analysis. Thematic analysis will be conducted. Three researchers will work independently (i.e., Qi, Ellaisha, and Carol) to identify the key themes based on the first few transcripts with a subsequent discussion to refine the code themes, and then to develop the code-book. Disagreement will be solved through further discussion to achieve consensus. All qualitative analysis will be done through NVivo V.11.

G5 Please provide a list of relevant references

Abdin, E., M. Subramaniam, E. Achilla, S. A. Chong, J. A. Vaingankar, L. Picco, R. Sambasivam, S. Pang, B. Y. Chua and L. L. Ng (2016). "The societal cost of dementia in Singapore: results from the WiSE study." Journal of Alzheimer's Disease 51(2): 439-449.Alzheimer's Association and National Alliance for Caregiving (2004). Families Care: Alzheimer's Caregiving in the United States 2004.Brodaty, H. and M. Donkin (2009). "Family caregivers of people with dementia." Dialogues in clinical neuroscience 11(2): 217.Brown, E. L., N. Ruggiano, J. Li, P. J. Clarke, E. S. Kay and V. Hristidis (2017). "Smartphone-based health technologies for dementia care: Opportunities, challenges, and current practices." Journal of Applied Gerontology: 0733464817723088.Brown, E. L., N. Ruggiano, L. Roberts, V. Hristidis, K. L. Whiteman, J. Castro and T. F. Page (2016). "CareHeroes Web and Android™ Apps for Dementia Caregivers: A Feasibility Study." Research in Gerontological Nursing 9(4): 193-203.Butcher, H. K., P. A. Holkup and K. C. Buckwalter (2001). "The experience of caring for a family member with Alzheimer’s disease." Western Journal of Nursing Research 23(1): 33-55.Callan, J. A., G. J. Siegle, K. Abebe, B. Black, L. Martire, R. Schulz, C. Reynolds III and M. H. Hall (2016). "Feasibility of a pocket-PC based cognitive control intervention in dementia spousal caregivers." Aging amp; mental health 20(6): 575-582.Cohen, C. A., A. Colantonio and L. Vernich (2002). "Positive aspects of caregiving: rounding out the caregiver experience." International journal of geriatric psychiatry 17(2): 184-188.Devi, F., Q. Yuan, P. Wang, G. T. H. Tan, R. Roshan Goveas, L. L. Ng, S. A. Chong and M. Subramaniam (2020). "Positive aspect of caregiving among primary informal dementia caregivers in Singapore." PLOS ONE 15(8): e0237677.Eysenbach, G. and C.-E. Group (2011). "CONSORT-EHEALTH: improving and standardizing evaluation reports of Web-based and mobile health interventions." Journal of medical Internet research 13(4).Givens, J. L., C. Mezzacappa, T. Heeren, K. Yaffe and L. Fredman (2014). "Depressive symptoms among dementia caregivers: Role of mediating factors." The American Journal of Geriatric Psychiatry 22(5): 481-488.Grossman, M. R., D. K. Zak and E. M. Zelinski (2018). "Mobile Apps for Caregivers of Older Adults: Quantitative Content Analysis." JMIR mHealth and uHealth 6(7).Haro, J., K. Kahle-Wrobleski, G. Bruno, M. Belger, G. Dell’Agnello, R. Dodel, R. Jones, C. Reed, B. Vellas and A. Wimo (2014). "Analysis of burden in caregivers of people with Alzheimer’s disease using self-report and supervision hours." The journal of nutrition, health amp; aging 18(7): 677-684.Infocomm Media Development Authority of Singapore (2018). ANNUAL SURVEY ON INFOCOMM USAGE IN HOUSEHOLDS AND BY INDIVIDUALS FOR 2017. Singapore.Karanicolas, P. J., F. Farrokhyar and M. Bhandari (2010). "Blinding: Who, what, when, why, how?" Canadian journal of surgery 53(5): 345.Kramer, B. J. (1997). "Gain in the caregiving experience: Where are we? What next?" The Gerontologist 37(2): 218-232.Lazarus, R. S. and S. Folkman (1984). Stress, appraisal, and coping. New York, Springer.Meyerowitz-Katz, G., S. Ravi, L. Arnolda, X. Feng, G. Maberly and T. Astell-Burt (2020). "Rates of Attrition and Dropout in App-Based Interventions for Chronic Disease: Systematic Review and Meta-Analysis." Journal of medical Internet research 22(9): e20283-e20283.Mougias, A., A. Politis, M. Mougias, I. Kotrotsou, P. Skapinakis, D. Damigos and V. Mavreas (2015). "The burden of caring for patients with dementia and its predictors." Psychiatrike= Psychiatriki 26(1): 28-37.Pinquart, M. and S. Sörensen (2007). "Correlates of physical health of informal caregivers: a meta-analysis." The Journals of Gerontology Series B: Psychological Sciences and Social Sciences 62(2): P126-P137.Rathnayake, S., W. Moyle, C. Jones and P. Calleja (2018). "mHealth applications as an educational and supportive resource for family carers of people with dementia: An integrative review." Dementia: 1471301218768903.Reyes, A. K., J. E. Camargo and G. M. Díaz (2015). Design of a mobile application to support non-pharmacological therapies for people with Alzheimer disease. International Conference on Smart Health, Springer.Sallim, A. B., A. A. Sayampanathan, A. Cuttilan and R. C.-M. Ho (2015). "Prevalence of mental health disorders among caregivers of patients with Alzheimer disease." Journal of the American Medical Directors Association 16(12): 1034-1041.Schulz, R. and L. M. Martire (2004). "Family caregiving of persons with dementia: prevalence, health effects, and support strategies." The American journal of geriatric psychiatry 12(3): 240-249.Subramaniam, M., S. A. Chong, J. A. Vaingankar, E. Abdin, B. Y. Chua, H. C. Chua, G. K. Eng, D. Heng, S. B. Hia and W. Huang (2015). "Prevalence of dementia in people aged 60 years and above: results from the WiSE study." Journal of Alzheimer's Disease 45(4): 1127-1138.Tan, G. T. H., Q. Yuan, F. Devi, P. Wang, L. L. Ng, R. Goveas, S. A.

Chong and M. Subramaniam (2021). "Factors associated with caregiving self-efficacy among primary informal caregivers of persons with dementia in Singapore." BMC Geriatrics 21(1): 13.Whitehead, A. L., S. A. Julious, C. L. Cooper and M. J. Campbell (2016). "Estimating the sample size for a pilot randomised trial to minimise the overall trial sample size for the external pilot and main trial for a continuous outcome variable." Statistical methods in medical research 25(3): 1057-1073.WiSE team and Institute of Mental Health Research Division (2014). Dementia in Singapore: a report on the key findings. Singapore, Research Division, Institute of Mental Health.Xu, J., Y. Zhang, C. Qiu and F. Cheng (2017). "Global and regional economic costs of dementia: a systematic review." The Lancet 390: S47.Yuan, Q., G. T. H. Tan, P. Wang, F. Devi, R. Goveas, H. Magadi, L. L. Ng, S. A. Chong and M. Subramaniam (2021). "Combining a variable-centered and a person-centered analytical approach to caregiving burden – a holistic approach." BMC Geriatrics 21(1): 286.Yuan, Q., T. H. Tan, P. Wang, F. Devi, H. L. Ong, E. Abdin, M. Harish, R. Goveas, L. L. Ng, S. A. Chong and M. Subramaniam (2020). "Staging dementia based on caregiver reported patient symptoms: Implications from a latent class analysis." PLOS ONE 15(1): e0227857.Yuan, Q., P. Wang, T. H. Tan, F. Devi, D. Poremski, H. Magadi, R. Goveas, L. L. Ng, S. A. Chong and M. Subramaniam (2020). "Coping patterns among primary informal dementia caregivers in Singapore and its impact on caregivers - Implications of a latent class analysis." The Gerontologist.

G6 Please submit a copy of at least two relevant papers

Ref 1 Mobile Apps for Caregivers of Older Adults _ Quantitative Content Analysis.pdf

Ref 2 mHealth applications as an educational and supportive resource for family carers of people with dementia _ An integrative review.pdf

G7 Does this study have a Study Protocol?

○ Yes
◉ No

G8 What is the estimated time needed to conduct this study?

No. of Years 1 **and** **No. of Months 9**

G9 The PI is responsible for ensuring that all research participants give informed consent before enrolling into the study.* Click here for help

Please select all the applicable consent scenarios.

☑ **Informed Consent will be taken for study subjects.**
☐ **Waiver of Informed Consent is requested for study subjects.**
☐ **Community Consent will be taken.**

☐ **In addition, there will be use of anonymised data and/or biological materials from an independent third party for a specific study population.**

Please elaborate why a combination of both informed consent and waiver of consent is required, and which population(s) will require waiver of consent and which population(s) will be able to give informed consent.

H1. Identify all categories or groups, primary or secondary target, age range and total number to be enrolled (consented).* Click here for help Please state the target number of research subjects to be recruited for each study site, taking into account subject dropouts under the 'Maximum Total Enrolment Target'. Primary targets are those who either give consent or those who can only provide assent (e.g., minors). Secondary targets are those who provide data to supplement the primary target data (e.g., parents completing a questionnaire, teachers who supply information and data).

No.	Study Site (s)	Group / Population (e.g. Parents, Students, Residents, House Owners, Patients)	Primary or Secondary Target	Age Range (e.g. 6-12, 13 -21, 21-99)	Minimum Total Enrolment Target	Maximum Total Enrolment Target
1	Institute of M ental Health	Informal care givers of pers ons with dem entia	Primary	21-99	65	115

Total 65 115

H2 Please provide justification for selection of all the group/population indicated in Section H1

The current study aims to explore the feasibility and potential effectiveness of a mobile-based intervention on the mental health of local informal caregivers of persons with dementia. As such, caregivers of persons with dementia are the target population for this study.

H3 Please list the inclusion criteria. (Note: persons below the age of 21 who are not and were never married are considered minors in Singapore and would require parental consent prior to participation). * Click here for help

For the FGD, the inclusion criteria are1) aged 21 or above; 2) Singapore citizen or permanent resident; 3) key caregiver who is currently taking care of a PWD; 4) able to read, write, and speak in English. For the RCT phase and the follow-up qualitative interview phases, the inclusion criteria are1) aged 21 or above; 2) Singapore citizen or permanent resident; 3) key caregiver who is currently taking care of a PWD; 4) scores 4 and above using the 4-item screening version Zarit Burden Interview; 5) has sufficient skills in using mobile apps; 6) able to read, write, and speak in English.

H4 Please list the exclusion criteria. Please state clearly, if pregnant women will be excluded from the study.* Click here for help

Caregivers with the following conditions will be excluded from the current study1) pregnant;2) with vision or hearing problems;3) paid caregivers.

H5 Are there any recruitment restrictions based on the gender of the research participants?

◉ No
○ Yes

H6 Are there any recruitment restrictions based on the race of the research participants?

◉ No
○ Yes

H7 Does the study involve any of the vulnerable Research Participants ?

◉ No

○ Yes

H8 Is this study part of an international study?

◉ No
○ Yes

H9 Does the study involve any of the following?*

☐ Inpatient
☐ Outpatient
☑ Healthy Volunteers
☐ Not applicable
☐ Others (e.g. parents, students, residents, house owners)

I1

Please list all procedures, measures and analyses that will be performed in this study. If you are extracting data from existing record, please indicate the period of data that will be extracted (E.g. 1 Jan 2000 ï¿½31 Dec 2010)

In your list, indicate the procedures that are being done for non-research purposes (i.e Procedures that are performed as part of curriculum / is performed for diagnostic/standard clinical purposes).

The project will have three phases, including 1) app design and development, 2) intervention evaluation, and 3) seeking users' feedback.App design and development phase - during this phase, focus group discussions will be conducted by the study team members to collect feedback from potential users once the prototype of the app is ready. This information will be used to improve the app for another round before launching the final app during the intervention evaluation phase. The planned sample size is 15, however, the final sample will be determined by data saturation. The group size will vary from 4-6 in considering their usual heavy involvement in the daily caregiving and the fact that we only have a short window period to collect such information (2-4 weeks). The intervention evaluation phase - From this phase onwards, the final app will be provided to the users. In order to explore the feasibility and the potential effectiveness of the intervention (i.e., the final app), a pilot RCT with a waiting-list control condition will be conducted. The target sample size is 100 key dementia caregivers with relatively high baseline caregiver burden (scored 4 and above using the 4-item Screening Version Zarit Burden Interview (Bédard, Molloy et al. 2001)). Participants will be approached in IMH and satellite clinics, or referred by our collaborating clinicians. Public advertisement (e.g., online advertisement) will also be put up to reach potential participants. Once the participant agrees to participate, a study team member will go through the informed consent form with the participant either online via a video-conferencing platform such as zoom or in-person at a place convenient to the participant depending on the preference of the participant. After giving the signed consent, a screening session using the 4-item Zarit burden interview will be conducted. Participants with a score of 4 and above will be eligible and will be required to complete the baseline assessment directly. Block randomization with a block size of 4 will be used to allocate the caregivers into either the intervention (n=50) or the control conditions (n=50). An independent statistician who is not involved in the daily implementation will conduct the randomization, using a computer-generated random sequence table (i.e., AABB, ABAB, ABBA, BABA, BAAB, and BBAA). This is to ensure the allocation concealment. Caregivers in the intervention group will be instructed to use the app for at least 30 minutes per week over a period of 1 month. During this 1-month period, caregivers will be required to write about their positive thoughts and feelings on caregiving twice per week (i.e., one in the middle of the week, and the other one at the end of the week) using the 'Positive Reflections' feature, and this is the minimum requirement for the participants in the intervention group. For the networked peer support platform, users can post content, respond to others and get notifications when new interactions have taken place. Such interactions will provide natural triggers to bring users back to the platform and may as a result increase engagement (Fogg 2009). Meanwhile, an admin account will be kept by the research team to monitor the platform and ensure the overall supportive environment of the platform. Caregivers can use the app in ways that best fit their schedule and interests during the intervention period. Follow-up calls will be made by the study team to the intervention group participants every week to seek feedback, clarify potential confusion, and motivate them. Patient Health Questionnaire -9 (PHQ-9) will be used to assess the mental health status of users in the intervention group after week 2 to monitor their potential risk (and to report and provide necessary support if needed). In the meantime, caregivers in the control group will be placed on a waiting list for one month. After this one-month period, post-assessments will be conducted for both groups. Comparisons between the post-assessment and baseline assessment will be used to determine the potential effectiveness of the mobile app intervention. Usage data of the participants in the intervention group will be automatically documented by the system and will be analyzed to explore the relationship between usage patterns and interventional effects. Last but now least, participants in the waiting list control group will be granted access to the app as well after they finish post-assessment. However, this last step is more of an in-kind offer to the participants in the control group which they can refuse if they don't want to have such access. And it is not part of the research procedures, no data or further inquiries will be conducted during this step. Depressive symptoms will be measured by the 20-item Centre for Epidemiological Study Scale (CES-D) (Radloff 1977). Knowledge of

dementia will be measured by the 27-item Dementia Knowledge Assessment Scale (DKAS) (Annear, Toye et al. 2015). Caregiving self-efficacy will be measured by the 15-item Revised Scale for Caregiving Self-efficacy (Steffen, McKibbin et al. 2002). Coping strategy will be measured by the 28-item brief Coping Orientation to Problems Experienced (COPE) (Carver 1997). Positive aspects of caregiving will be measured by the 9-item Positive Aspects of Caregiving Scale (PAC) (Tarlow, Wisniewski et al. 2004). Caregiver burden will be assessed using the 22-item Zarit Burden Interview (ZBI) (Zarit, Reever et al. 1980). Anxiety will be assessed by the Generalized Anxiety Disorder 7-item scale (GAD-7) (Spitzer, Kroenke et al. 2006). Mental well-being will be measured by the 22-item Positive Mental Health instrument (PMH) (Vaingankar, Subramaniam et al. 2014). For the RCT, results will be reported following the Consolidated Standards of Reporting Trials (CONSORT) statement regarding eHealth (Eysenbach and Group 2011). Group information will not be disclosed to the statistician who is in charge of the final analysis, this is to blind the data analyst to reduce bias (Karanicolas, Farrokhyar et al. 2010). The primary analysis will follow the intention-to-treat principle and compare the outcome measures between the intervention vs. control groups. In addition, per-protocol analyses will also be conducted. More specifically, descriptive statistics will be used to describe the participants' profiles and their characteristics. The Mann–Whitney U-test will be used to assess any significant differences in the demographic data and clinical characteristics of the two groups. A multivariate repeated measured ANOVA will be performed to examine the differences between the two groups over time, with the adjustment of potential baseline differences. Effect size (i.e. Cohen's d) will also be calculated. All analysis will be conducted through SPSS V21 or SAS 9.4.The intervention and the assessments are both straightforward and closely related to the study aims, no other tests or assessments will be conducted. As such, we expect that there will be no possibility of incidental findings which have potential health or reproductive importance. Seeking users' feedback phase - after the RCT, a follow-up qualitative interview study with around 20 participants will be conducted to seek users' feedback on the mobile app and to identify features/aspects which can be further improved (final sample size also depends on saturation). No particular selection process will be employed; instead, participants in the intervention group will be invited as soon as they finish the post-assessment. This qualitative interview study will enable a more in-depth evaluation of the app and information generated can be used to further improve the program in potential future studies. For this qualitative sub-study, the interviews will be conducted by the study team members, and will be audio-recorded and transcribed prior to the analysis. Thematic analysis will be conducted. Three researchers will work independently (i.e., Qi, Ellaisha, and Carol) to identify the key themes based on the first few transcripts with a subsequent discussion to refine the code themes, and then to develop the code-book. Disagreement will be solved through further discussion to achieve consensus. All qualitative analysis will be done through NVivo V.11.

I2 Please attach the Data Collection Form (if applicable), Questionnaire Form (if applicable), Interview/Focus Group Discussion Guide (if applicable) and/or List of Variables that will be extracted from medical record/database (if applicable)* Click here for help

Dementia app Questionnaire _ baseline assessment.doc

Dementia app Questionnaire _ post assessment.doc

Table 1 Interview Guide_v1.1.docx

Focus Group Discussion Interview Guide v1 _ 9 March 22.docx

clean_PHQ9 id date 08.03.pdf

I3 Discuss the potential difficulties and limitations of the proposed procedures and alternative approaches to achieve the aims.

Due to the current COVID situation, caregivers may refuse to join the study due to safety concerns. As such, we will also offer to go through the informed consent and complete the discussion/assessments/interviews online via videoconferencing. This is also to maximize our recruitment.

◉ No
○ Yes

J1 How will potential Research Participants be identified? (Please tick all the applicable boxes)

☑ Referral by attending healthcare professional

☑ Persons with dependent relationship with study team (e.g. doctor-patient, employee-employer, head-subordinate, student-teacher, departmental staff relationship)

Please state clearly the dependent relationship of the study team members with the research participants. In addition, please describe how the study team will manage the dependent relationship to prevent coercion or undue influence (e.g. informed consent will not be taken by the primary physician of the participants, sufficent time will be given to the participants for their consideration in the research study and voluntary participation will be emphasized to the participants)

For some informal caregivers, their relatives with dementia might be seeking treatment from the clinician who is part of the research team. In this case, the clinician could refer the caregivers to us. Please note that the clinician will not be involved in the consent-taking and interview process. The potential participants will be followed up by other research team members. This is to avoid perceived coercion.Due to the slow recruitment, the principal investigator or the designated staff will go to CGH to receive the collaborating clinician's referral. No other procedures will be conducted in CGH.

☐ Databases

☑ Other methods of identifying potential research participants

Please specify

There are a few different ways to identify the research participants:1) the study team will also actively approach potential participants in the outpatient clinics and ask them if they are looking after a person with dementia to avoid approaching non-dementia caregivers;2) our collaborating clinician may refer informal caregivers of some of their patients to us, and these potential participants will be followed up by the study team members;3) since we will put up the recruitment advertisement online, potential participants who see this advertisement might contact us, we will follow up onwards;4) we may also actively contact some participants from two previous studies (DSRB ref - 2016/00921 amp; 2018/01069) who were agreeable to be contacted for future studies to check if they are willing to join;5) participants will also be asked to refer their peers to us.

J2 Will there be direct contact with Research Participants?

○ **No**

◉ **Yes**

(i) Who will make the first contact with Research Participants ?

For potential participants referred by the clinicians, the clinician will be the first contact point, and then the potential participant will be followed up by the study team members.For all other recruitment methods, one member of the study team or dedicated research assistant will make contact with the research participants.

(ii) How will the Research Participants be contacted ?* Click here for help

1) For participants regularly accompanying the PWDs to visit the IMH satellite clinics, we will approach them face-to-face in the clinics.2) In some circumstances, our collaborating clinician or the participants themselves may refer eligible participants to us. Before we get the referrals, we will emphasize to them to seek potential participants' approval first. The potential participants will be the ones to provide the contact details to pass on to the study team. We will only approach the referred caregivers if they agree to be contacted by us. In this case, we may call or SMS the referred caregivers to follow up. 3) For potential participants who contact us after they see the online recruitment advertisement, the study team member will follow up with them through phone calls. 4) For caregivers who agreed to be contacted for future studies in our previous research projects (DSRB ref - 2016/00921 amp; 2018/01069), we will also contact them through phone call or SMS to check if they are willing to join the current study. 5)

Caregivers who are referred by their peers will be followed up by the study team via calls or SMS if they are willing to join the study.

(iii) Will any advertising / recruitment materials (for e.g. posters/brochures/advertisements/ telephone/email script) be used to recruit Research Participants?* Click here for help

○ No
◉ Yes

Please tick all the applicable types of advertising / recruitment materials that will be used in this study.

☑ Posters
☐ Brochures
☐ Advertisements in Newspapers / Magazines / Publications
☐ Advertisements on Radio / TV.
☐ Letter of Invitation to potential research participants. Letter of Invitation refers to email, letters or any form of documents used as part of the recruitment strategy, with the intention of inviting the research participants to participate in the study.
☐ Letter to Doctors requesting for referrals.
☐ Other types of materials will be used.

Please state where the advertising/recruitment material selected will be placed and frequency (if applicable) of advertisement

The recruitment flyer might be used in several ways:1) it might be put in the clinics as posters, to enable potential participants who are interested in this study to contact us;2) It might also be put up on social media platforms such as Facebook, Instagram, and Linkedin. We will create specific accounts for the project itself to upload such info. In the meantime, we will approach our corp com to see if possible to put on the official social media accounts of IMH. Last but not least, we will also approach voluntary welfare organizations such as Caregiver Alliance or Alzheimer's Disease Association to see if possible to put on their social media accounts as well. 3) the clinician might also keep copies of the recruitment flyers so that they can distribute them to the caregivers who are potential research participants.

Please attach a copy of all advertising / recruitment materials

Recruitment flyer _v1.3 _ 3 Jun 2022.docx

(iv) Will any other recruitment strategies be used? (Eg. Talks in public places, societies etc.)

◉ No
○ Yes

(v) Please indicate the length of time of the Research Participant's direct involvement in the study.E.g For completion of surveys, focus group discussion, taste evaluation, clinical visits, examinations etc. (If applicable)

For the focus group discussion during the app design and development phase, the discussion will take 1-2 hours, and it will comprise the following procedures:1) obtain written informed consent from all the participants;2) conducting the discussion;3) disbursement of inconvenience fees.For participants in both groups during the pilot RCT, the study will involve completing two assessments which each will last for 1-2 hours. The assessment will comprise the following activities : 1) obtain written informed consent from the participant (only for the baseline assessment);2) complete the assessment;3) disbursement of inconvenience fee.For participants in the intervention group, they will be required to use the app developed by us for one month. They will be advised to use it for at least

30 minutes per week and complete two positive reflection entries, but they will decide how they will use it in reality. The study team will call them on a weekly basis to solve any issues they face while using the app and to motivate them. PHQ-9 will be administered to the participants in the intervention group after week 2 to monitor their mental health, this will take less than 10 minutes. We will also conduct a follow-up interview among some of the participants in the intervention group (the final sample depends on the saturation). This interview will take 30-60 minutes, and it will comprise the following procedures:1) obtain written informed consent from the participant;2) conducting the interview;3) disbursement of inconvenience fee.We may also contact the interview participants within three months upon the completion of the interviews in case we missed anything from the recording. Such contact will be done via phone call and normally won't last for more than 10 minutes.

K1 Describe when the consent process will take place with the potential research participant/legally acceptable representative, including the time provided for him/her to consider his/her participation in the study.* Click here for help

For the FGD - written informed consent will be obtained from the participant first. After completing the consent session, the participants will be instructed to download and install the mobile app and explore it. The FGD will be scheduled within one week after obtaining the written consent.For the pilot-RCT and the follow-up qualitative interviews, written informed consent will be obtained from each participant on the day of the discussion, the baseline assessment, and the follow-up interview, prior to the commencement. The participants will be given as much time as necessary to decide their participation. During the initial approach/ contact, they will also be given information about the study, their queries answers and they will be asked verbally if they are willing to participate before they are enrolled in the project.

K2 Where will consent be taken (e.g. room, ward, outpatient clinic etc)? How will privacy, freedom from intrusion and comfort be ensured?* Click here for help

Consent will be obtained at the site of the discussion/assessment/interview if participants choose to do it face-to-face, and will be obtained online via Zoom if the participants choose to do it online. For face-to-face consent taking, we will ensure that there will be an individual room for the discussion/assessments/ interview without the intrusion of other family members or people so that the participants can think without any influence of others and take time to consider their responses. For the online consent process, we will remind the potential participants to do it in a private location to ensure privacy and confidentiality.

K3 Who will take consent from potential research participants/legally acceptable representatives (e.g. PI, Co-Investigators etc)?* Click here for help

The PI or other study team member who is conducting the assessment/interview will take consent from the participants.

K4 Besides the Informed Consent Form, will any other materials or documents be used to explain the study to potential research participants/legally acceptable representatives? (eg. scripts, handouts, brochures, videos, logs, etc).

- ◉ **No**
- ○ **Yes**

K5 Will Research Participants receive any monetary payments (including transportation allowances) or gifts for their participation in the study?* Click here for help

- ○ No
- ◉ Yes

Please describe:

For the focus group discussion, participants will receive SGD 30 if they complete the discussion. For the RCT, participants will receive SGD 25 if they complete the baseline assessment, and another SGD 35 if they complete the post-assessment. For the follow-up qualitative interview, participants will receive SGD 30 if they complete the interview.

K6 Will consent be documented in the form of a written and signed Informed Consent Form?

- ◉ Yes, all research participants/legally acceptable representatives will be given a copy of the Informed Consent Form.

Please attach a copy of the Information Sheet and Consent Form.*

3 Demenia app project interview phase ICF _ v1.2 _ 22 Apr 2022 - Clean.doc

3 Demenia app project interview phase ICF _ v1.2 _ 22 Apr 2022.doc

1 Demenia app project FGD ICF_ v1.3 5 Aug 2022.doc

1 Demenia app project FGD ICF_ v1.3 5 Aug 2022 - Clean.doc

2 Demenia app project evaluation phase ICF _ v1.7 _5 Aug 2022.doc

2 Demenia app project evaluation phase ICF _ v1.7 _5 Aug 2022 - Clean.doc

○ No, Consent will not be documented. (E.g. verbal consent).

K7 Consent Language Will the study enrol non-English speaking research participants/legally acceptable representatives?

◉ No

Please explain why.

This is just a preliminary study which only aims to explore the feasibility and potential effectiveness. Moreover, we only have a limited budget in app development and can only afford the app in the English language.

○ Yes

K8 Do you have any additional comments regarding the Informed Consent process?

○ No

◉ Yes

Please elaborate:

A bit more details on the online content taking process:The video-conferencing platform Zoom will be used to obtain consent from the participants online. One day before the consent-taking session, a soft copy of the ICF together with a document on how to sign a file via an electronic device will be sent to the participant so that they can review these documents in advance.A study team member and witness will be in the consent-taking process alongside the participant. The presence of the witness is to ensure the ICF process is conducted properly. At the start of the Zoom meeting, participants will be requested to show photo identification of themselves to prove their identity. Similarly, the study team will show their IMH staff identification to prove their identities. The study team member will go through the ICF with the participant. After the study team member who is taking the consent clears all doubts of the participant and the participant verbally agrees to sign the ICF to continue the study, the study team member will send an ICF to the participant via email with the witness being in the CC list. The signature will be done in the sequence of 'participant – witness – consent taker'. Adobe Acrobat Reader (PC) and Adobe Fill amp; Sign (mobile) will be used to obtain electronic signatures, and the individual who is signing will be required to turn on the share screen feature of Zoom to enable others to verify the process. The consent taker will save the ICF into a read-only version after he/she puts in his/her signature, this is to prevent any unauthorized edition on the signed ICF file. The consent taker will send this read-only ICF to the participant afterward, for his/her records. It will also be kept in the secure drive which is only accessible via IMH intranet, and only eligible study team members will be granted the access.

K9: Will a witness be present during the consent process? For studies that are under the purview of the HBRA, consent must be obtained in the presence of a witness, unless the requirements for exemption are met.*Click here for help

◉ Yes

○ No

K10 If community consent is required for this study, please describe how it will be obtained. Please attach copies of documents to be used in this community consent process.*Click here for help

NA

Identify the risk(s) to individual subject and group/population according to the respective categories. Then provide details on the steps that will be taken to overcome or minimize the risk (if any).* Click here for help

- ☐ M1 Economic / Financial Risk
- ☐ M2 Legal Risk
- ☐ M3 Physical Risk
- ☑ M4 Psychological Risk

Section

Group	Identify the risk & steps to overcome / minimise the risk	Probability (High /Low)	Magnitude (High / Low)
Individual Research Participant	For the assessments, participants may feel some discomfort while answering questions related to their emotional and psychological health. They will be given choices including not answering any questions that make them uncomfortable, taking a break and continuing later on, or opt-out at any point of the study. For the FGD/ interview, it is possible that the participants might	Low	Low

	experience fatigue during the session/ interview. We will tell them if this happens, please feel free to request a break by informing the researcher. For questions or topics that the participants are uncomfortable addressing, they can choose to skip through or withdraw from the study at any time. Last but not least, the interviewer will look out for signs of distress among the participants during the assessments and/or interview/ FGD. These interviewers are either psychology-trained or those with many years of experience of working with persons with mental illness. They will provide onsite assistance to the distressed participants to deescalate	

	their stress levels. Extremely distressed participants will be referred to our collaborating clinicians for immediate help (given that they are willing to). A flyer with necessary helplines will still be provided to those with signs of distress and they will also be asked to follow up with their family physician for help.		
**Group / Population**	NA	**Not applicable**	**Not applicable**

☑ M5 Social Risk

Section			
**Group**<br>**Individual Research Participant**	**Identify the risk & steps to overcome / minimise the risk**	**Probability (High /Low)**<br>**Low**	**Magnitude (High / Low)**<br>**Low**
	As the app has some social media attributes (i.e., the virtual communities of the online peer support forum), it's possible that there might be social		

	risk. We have implemented the following mitigating measures including:1) a 'report' function to enable users to report inappropriate posts – after being reported, a notification email will be sent to the system admin immediately, and the system admin will review the reported item in the admin portal to decide if to edit/delete the post/ thread; 2) the system admin will review the forum on a daily base to ensure there is no inappropriate posts/threads in the forum (edit/remove the posts/ threads if needed);3) a block-list function is available to the users so that they can block other users which they don't want to interact with.		
**Group / Population**	NA	**Not applicable**	**Not applicable**

M6 Discuss any potential benefits to the individual Research Participants and/or the population of Research Participants and/or to the society that justify involvement of Research Participants in this study.

There is no assurance that the participants might benefit from participation in this study or not. However, their participation in the study may add to the knowledge about the use of this mobile app to support caregivers of persons with dementia in the local context.

M7 Is it appropriate for your research to have a monitoring plan to periodically assess the data to ensure the safety of Research Participants or to ensure negative outcomes do not occur (e.g. physiological stress, employment termination etc)* Click here for help

○ No
◉ Yes

(a) Who performs the data and safety monitoring?

◉ Principal Investigator and/ or Study Team
○ Data Safety Monitoring Board (DSMB) (Please submit the DSMB charter)
○ Others

(a) Describe the steps that you will be taking to assure that Research Participants are protected. You may attach a copy of the plan

null

(b) Please state the Safety Monitoring plan, i.e. frequency of review (e.g. daily, weekly, quarterly) and type of data (e.g. adverse events/serious adverse events) will be monitored.* Click here for help

Adverse events (i.e., depression) will be monitored. We will call the participants in the intervention group after week 2 to assess their depression status using Patient Health Questionnaire (PHQ-9), participants with a score above 20 will be considered as at severe risk and will be asked to seek treatment with their family physician. These cases will also be reported to DSRB as adverse events.Causal relationships will be assessed following the three criteria for determining causal relationships, including 1) Empirical association – empirically these events should be related to the study or study procedures;2) Temporal relationship – the event should happen after the intervention is introduced;3) Nonspuriousness – no other possible reasons or explanationsThe study team member conducts the recruitment or the PI will be responsible for assessing the events; the PI will be responsible for managing the events.

(c) Please state the Data Monitoring plan, i.e. frequency of review (e.g. daily, weekly, quarterly), how data integrity is assured.

The questionnaire will be checked for discrepancies by the person who administers the interview as soon as the completed questionnaire is collected. The data will be double entered on a monthly basis to ensure data integrity and quality.For FGD and qualitative interview data, the interview process will be audio-recorded. The recorded file will be transcribed verbatim immediately after the interview, and be crossed-checked by another study team member.Data confidentiality will be monitored on a monthly base – the principal investigator will check the hard copy data collection forms to ensure they are all under locked conditions and only accessible to the authorized study team members. We will also call up a monthly meeting with the vendor to let them report the status of the app and the server. This is to monitor the data security status. iHIS will be involved if needed.

N1 In general, to protect the Research Participant's confidentiality, research data should be coded, and the links between the Research Participant's identifiers and the codes should be stored separately from the research data.

Please state how the research data will be protected to ensure confidentiality and security.

- ☑ For hardcopy data, they will be stored in designated locked cabinet(s) or room(s) that are accessible to authorized study personnel only.
- ☑ For electronic data, they will be stored on in a secured computer that is password-protected. The databases will not contain subject identifiers and the data linking subject identifiers and the subject identification codes will be stored separately. When portable media (e.g. CD, USB drives etc.) are used to store the data, subject identifiers are stored separately.

N2 Describe who will have access to the research data.* Click here for help

Only authorized study personnel who are also the study team members will have access to electronic data. Period audits will be conducted to monitor adherence to these data safeguarding procedures.

N3 Will research data be released and shared with individuals or entities outside the institution? If yes, please ensure that only coded data is shared to protect data confidentiality.

- ◉ No
- ○ Yes

N4 Will the research data be used for future research after the study is completed?* Click here for help

- ◉ No, the research data will be destroyed after it has been stored for 6 years or minimum duration of retention period as specified by your institutional policy, whichever that is longer.
- ○ Yes, the research data will be used for future research. Please register a standing database with DSRB only once the study has completed.

N5 Will any part of the study procedures be recorded on audiotape, film/video, or other electronic medium (excluding non-identifiable images such as MRI/ X-Ray/ CT)?

- ○ No
- ◉ Yes

(a) Please describe the contents of the recording (e.g. audio-recording of interview/ focus group discussion, images of facial features, etc.).

All interviews including the FGD and in-depth interviews will be audio recorded to assist the study team in accurately capturing what participants have said during the interviews.

(b) What is the medium (audio tape / video etc) used for recording?

An audio recorder will be used.

(c) Explain how the recorded information will be used in the study.

The recordings will be used for transcription. For analysis purposes, a separate folder that can be accessed by all members of the research team will be created. A copy of de-identified transcripts will be in this folder.

(d) For how long and where will the recording medium be stored? Who will have access, and how will access be controlled and monitored?

The audio recordings will be stored in a dedicated research server in folders that are accessed controlled. Recordings will then be subsequently deleted after ensuring that transcriptions are as accurate as possible. Only investigators involved in transcription and quality checks for accuracy will have access to this access-controlled folder. Periodic audits will be conducted to monitor adherence to these data safeguarding procedures.

(e) How will the recording medium be disposed?

The recording is only stored on secure servers at IMH; it will be deleted from these servers upon the completion of the interview and the transcription.

O1 Will any biological materials (such as blood or tissue) be used as part of the study?

- (●) No
- (○) Yes

P1 Will research results be disseminated to the community? * (For community-based participatory research, the researchers should consider if the results should be disseminated to the community)

- ◉ No
- ○ Yes

P2 Will research results raise any community concern?

- ◉ No
- ○ Yes

Please ensure that the Curriculum Vitae is accurate and up to date.* Click here for help

No.	Study Site	Name	Study Role	CV
1	Institute of Mental H ealth	Dr Qi YUAN	PI	2 Resume_YUAN Qi_ 2018.pdf 03-Oct-201
2	Institute of Mental H ealth	Dr Mythily Subraman iam	Co-Investigator	CV_Mythily.doc 20-O ct-201
3	Institute of Mental H ealth	Dr Edimansyah Abdin	Co-Investigator	CV-Edimansyah Abdi n (updated july 2021) .doc 28-Jul-202
4	Institute of Mental H ealth	Ms ZHANG YUNJUE	Co-Investigator	Zhang Yunjue_CV.pd f 24-Apr-202
5	Institute of Mental H ealth	Ms Ellaisha Binte Sam ari	Co-Investigator	Ellaisha CV.docx 08-Oct-201
6	Institute of Mental H ealth	Dr Richard Roshan G oveas	Co-Investigator	Short CV for NTU.do c 24-Sep-201
7	Institute of Mental H ealth	Dr NG LI LING	Co-Investigator	CV full 2017 CGH.NL L.doc 21-Jun-201
8	Institute of Mental H ealth	Mr LIU LEI	Co-Investigator	CV_Liu Lei_2023_IM H.pdf 02-Mar-202
9	Institute of Mental H ealth	Ms Yun Ting Lee	Co-Investigator	Lee Yun Ting_CV.pdf 11-Dec-202

Your DSRB Application is now complete and ready for submission.

Principal Investigator's Declaration

I will not initiate this study until I have received approval notification from the DSRB and all applicable regulatory authorities.

I will not initiate any change in the study protocol without prior written approval from the DSRB, except when it is necessary to reduce or eliminate any immediate risks to the Research Participants. Thereafter, I will submit the proposed amendment to the DSRB and all applicable regulatory authorities for approval.

I will promptly report any unexpected or serious adverse events, unanticipated problems or incidents that may occur in the course of this study.

I will maintain all relevant documents and recognise that the DSRB staff and applicable regulatory authorities may inspect these records.

I understand that failure to comply with all applicable regulations, institutional and DSRB policies and requirements may result in the suspension or termination of this study.

I declare that there are no existing or potential conflicts of interest for any of the investigators participating in this study.

By checking the "I agree" box, you confirm that you have read, understood and accept the Principal Investigator's Declaration

☑ **I have read and agree to the above declaration.**

Principal Investigator: Qi YUAN
