# Supplementary material for: Evaluating a mobile-based intervention to promote the mental health of informal dementia caregivers in Singapore: Study protocol for a pilot two-armed randomised controlled trial
Source: PLoS One. 2024 Jun 24;19(6):e0305729. doi: 10.1371/journal.pone.0305729 (PMC11195940; doi:10.1371/journal.pone.0305729)
Supplement: S3 File — (PDF) [file pone.0305729.s003.pdf]

National Healthcare Group
Adding years of healthy life

3 Fusionopolis Link
#03-08 Nexus@one-north
Singapore 138543
Tel: 6496 6600 Fax:6486 6870
www.nhg.com.sg
RCB No. 200002150H

NHG DSRB Ref: **2022/00029**

15 August 2022

Dr Yuan Qi

Department of Research Division

Institute of Mental Health

Dear Dr Yuan

NHG DOMAIN SPECIFIC REVIEW BOARD (DSRB) APPROVAL

STUDY TITLE: Design and Pilot-test of an Innovative Mobile-based Intervention to Promote Mental Health of Informal Dementia Caregivers

We are pleased to inform you that the NHG Domain Specific Review Board has approved the application as titled above to be conducted in **Institute of Mental Health**.

The approval period is from **15 August 2022** to **14 August 2023**. The NHG DSRB reference number for this study is **2022/00029**. Please use this reference number for all future correspondence.

Please note that this is a human biomedical research that is regulated by the Human Biomedical Research Act (HBRA) and researchers are required by law to comply with all the relevant regulatory requirements of the HBRA.

Where stipulated by the Research Institution, Principal Investigators and study team members must complete the HBR minimum training prior to commencement of study involvement.

In accordance with the NHG Data Access & Sharing Policy, NHG investigators and study team members who require access to data that is not collected or created directly by the research team from research subjects or research materials (e.g. through interviews, surveys or experiments) should consult the relevant Data Exchange Office (DXO) to ascertain if a data access request should be raised. The NHG Institutional and Cluster DXO's details can be found at https://mynhg.nhg.com.sg/clst/nhgdxo/Pages/default.aspx.

The documents reviewed are:

a) NHG DSRB Application Form: **Version No. 1**
b) Baseline Questionnaire: Version 1.0 dated 27 October 2021
c) Post Questionnaire: Version 1.0 dated 27 October 2021
d) Interview guide: Version 1.1 dated 04 January 2022
e) Focus Group Discussion Interview Guide: Version 1.0 dated 09 March 2022
f) Patient Health Questionnaire (PHQ-9): Version dated 1999
g) Recruitment flyer: Version 1.3 dated 03 June 2022
h) FGD Informed Consent Form: Version 1.3 dated 05 August 2022
i) Interview Informed Consent Form: Version 1.2 dated 22 April 2022
j) Evaluation Informed Consent Form: Version 1.7 dated 05 August 2022
k) Screenshot of APP prototype: Version 1 dated 17 March 2022

Continued approval is conditional upon your compliance with the following requirements:

1. Only the approved Informed Consent Form should be used. It must be signed by each subject prior to initiation of any protocol procedures. In addition, each subject should be given a copy of the signed consent form.

2. Translated Informed Consent Forms (fully translated and short consent forms) and other translated documents are not required to be submitted to DSRB. It is the responsibility of the Principal Investigator to ensure that the translations for any document are an accurate reflection of the original approved content and to maintain the certification/documentation of the translations.

3. No deviation from or changes to the study should be implemented without documented approval from the NHG DSRB, except where necessary to eliminate apparent immediate hazard(s) to the study subjects.

4. Any deviation from or changes to the study to eliminate an immediate hazard should be promptly reported to the NHG DSRB within seven calendar days.

5. Please note that for studies requiring CTA/CTN/CTC, apart from the approval from NHG DSRB, no deviation from, or changes of the Research Protocol and Informed Consent Form should be implemented without documented approval from the Health Sciences Authority unless otherwise advised by the Health Sciences Authority.

6. Please submit the following to the NHG DSRB:

   a. All Unanticipated Problems Involving Risk To Subjects Or Others (UPIRTSOs) must be reported to the NHG DSRB. For more than minimal risk studies, all problems involving local deaths **must be reported as soon as possible**, but not later than 7 calendar days after first knowledge by the Investigator, regardless of the causality and expectedness of the death event, and any additional relevant information about the death should be reported within 8 calendar days of making the initial report. For no more than minimal risk studies, only problems involving local deaths that are related or possibly related to the study **must be reported as soon as possible**, but not later than 7 calendar days after first knowledge by the Investigator, and any additional relevant information about the death should be reported within 8 calendar days of making the initial report. For problems which are life threatening, it **must be reported as soon as possible**, but not later than 7 calendar days after first knowledge by the investigator, and any additional relevant information about the problems should be reported within 8 calendar days of making the initial report. All other problems that fulfil the UPIRTSOs reporting criteria **must be reported as soon as possible** but not later than 15 calendar days after first knowledge by the Investigator.

   b. Report(s) on any new information that may adversely affect the safety of the subject or the conduct of the study.

   c. NHG DSRB Study Status Report Form – this is to be submitted 4 to 6 weeks prior to expiry of the approval period. The study cannot continue beyond **14 August 2023** until approval is renewed by the NHG DSRB.

   d. Study completion – this is to be submitted using the NHG DSRB Study Status Report Form within 4 to 6 weeks of study completion.

With the enactment of the Human Biomedical Research Act, Health Products Act, Medicines Act and their subsidiary legislations, Principal Investigators are reminded to ensure that their research complies with the regulatory requirements stipulated in the applicable Acts. Contraventions under any of these Acts are criminal offences and would result in fines or imprisonment or both, subject to the nature of the offence.

NHG sites of studies regulated under the Human Biomedical Research Act will be subjected to the NHG Monitoring Programme. For Full Board studies, this will include on-site monitoring activities.

Established since May 2006, the NHG Research Quality Management (RQM) Programme seeks to promote the responsible conduct of research in a research culture with high ethical standards, identify potential systemic weaknesses and make recommendations for continual improvement. Hence, this

research study may be randomly selected for a review by the Research Quality Management (RQM) team. For more information, please visit www.research.nhg.com.sg.

The NHG DSRB operates in accordance to the ICH GCP and all applicable laws and regulations.

Yours Sincerely

A/Prof Adeline Seow

Chairperson

NHG Domain Specific Review Board F1

Cc: Institutional Representative, IMH

c/o Research Division, IMH

Departmental Representative of Research Division, IMH

(This is an electronic-generated letter. No signature is required.)
